# Biomarkers of post-acute infection syndrome: a systematic literature review

**DOI:** 10.3389/fimmu.2026.1741761

**Published:** 2026-06-30

**Authors:** Katharina Wendt, Maximilian Schieck, Christian Gille, Michael Marschollek, Thomas Illig, Dominik Wolff, Sarah Nee

**Affiliations:** 1 Peter L. Reichertz Institute for Medical Informatics of Technical University (TU) Braunschweig and Hannover Medical School, Hannover Medical School, Hannover, Germany; 2 Hannover Unified Biobank, Hannover Medical School, Hannover, Germany; 3 CAIMed - Lower Saxony Center for Artificial Intelligence and Causal Methods in Medicine, Hannover, Germany; 4 Department of Neonatology, Heidelberg University Children’s Hospital, Heidelberg, Germany

**Keywords:** biomarker, long-COVID-19, ME/CFS, omics, PAIS, post-COVID

## Abstract

**Background:**

Post-acute infection syndrome (PAIS) remained underrecognized before the COVID-19 pandemic, which further increased exposure by introducing a novel global cause. The global burden of post-acute COVID syndrome (PACS) and myalgic encephalomyelitis/chronic fatigue syndrome (ME/CFS) alone is estimated at several tens of millions affected worldwide. Biomarker discovery is central to improving PAIS diagnosis and may provide therapeutic targets. This review summarizes current knowledge on biomarkers for PAIS, including PACS and ME/CFS.

**Methods:**

A systematic literature search was conducted in PubMed and Web of Science. Inclusion criteria were: (1) studies including PAIS patients; (2) reporting laboratory or omics biomarkers; and (3) investigating biomarkers or pathomechanisms of PAIS. Although Guillain-Barré syndrome (GBS) is not PAIS, we have included it as a separate mechanistic comparator due to its prevalence in search results and its clinical and immunological similarities to PAIS.

**Results:**

A total of 142 studies analyzing PAIS biomarkers were included. GBS was analyzed separately and later compared with the other results. Overall, the reviewed studies employed heterogeneous approaches. While similar types of data were frequently investigated, analytical methods varied and often focused only on a subset of molecules. The results indicate that amino acid, energy, and lipid metabolism, microbiome, mitochondrial stress, and miRNA networks are affected. All pathways are connected via NF-κB.

**Discussion:**

PAIS is a multisystem disorder rooted in persistent immune activation, metabolic reprogramming, and systemic inflammation, driven not by active viral infection, but by dysregulated host responses. The NF-κB pathway serves as a unifying hub, connecting molecular, cellular, and clinical phenotypes. Our framework enables a shift from symptom-based to mechanism-based classification, paving the way for biologically grounded interventions.

**Conclusion:**

This review synthesizes a broad spectrum of biomarkers in PAIS, integrating findings across pathogens and molecular levels rather than restricting to individual conditions or symptom clusters. This study highlights the differences and commonalities among pathogens and diseases that lead to post-acute sequelae, fills a critical knowledge gap, and provides a foundation for future research and clinical practice. Future studies incorporating multi-omics approaches, longitudinal designs, and larger patient cohorts are needed to validate specific biomarkers and advance the understanding of PAIS.

## 1 Introduction

Post-acute infection syndrome (PAIS) refers to a complex of persistent symptoms that continue after an acute infection, without an otherwise identifiable cause (1). They can be triggered by a wide range of pathogens, including viruses, bacteria, fungi, and protozoa, with influenza viruses, SARS-CoV-2, and Epstein-Barr virus (EBV) among the most common. Additional infectious agents, such as herpesviruses, malaria, or *Coxiella burnetii* (Q fever), have also been described. Still, noninfectious cofactors, such as surgery or severe psychological stress, may function as modulators if preceded by infection (2). Despite their heterogeneity, all PAIS share the hallmarks of long-term health consequences and represent a growing medical and societal challenge.

Several clinical entities are considered part of the PAIS spectrum. Multiple terms, including long COVID syndrome, post-acute COVID-19 syndrome (PACS), and post-acute sequelae of COVID-19 (PASC), refer to conditions resulting from SARS-CoV-2 infection. Although PACS and PASC typically emphasize later time points than long-COVID, they are frequently used interchangeably (3). In this review, we use PACS as an umbrella term encompassing the various definitions. In contrast to PACS, resulting explicitly from SARS-CoV-2 infection, myalgic encephalomyelitis/chronic fatigue syndrome (ME/CFS) is associated with a variety of infectious triggers. While the global burden of all PAIS is difficult to determine, PACS alone affects an estimated 65 million, and ME/CFS approximately 20 million individuals worldwide (4, 5). Diagnostic accuracy is hampered by overlapping symptoms within and across syndromes, with misdiagnosis rates of ME/CFS estimated at 80-90% (6). A defining symptom of ME/CFS is post-exertional malaise (PEM), also known as post-exertional symptom exacerbation. Even minor physical or mental exercise can worsen other symptoms, including fatigue, difficulties with sleep and cognitive function, and pain. This exacerbation of symptoms can occur up to 3 days after activity and can last several months, significantly impacting the ability to participate in day-to-day life (7). While PEM can be objectively assessed via hand-grip strength testing, diagnosis of PAIS still relies on exclusion (8, 9). Although several diagnostic frameworks, such as the UK’s National Health Service (NHS) diagnostic and treatment guidelines for myalgic encephalomyelitis/chronic fatigue syndrome (ME/CFS) (NICE) (10), the Canadian Consensus Criteria (CCC) (11), the Institute of Medicine (IOM, United States) guidelines (12), and the D-A-CH (Germany, Austria, Switzerland) consensus statement (6) have been developed to provide valuable support for clinical evaluation, diagnosis of PAIS still lacks reliable diagnostic tests and biomarkers, and the pathogenesis of PAIS remains incompletely understood. Current hypotheses focus on the key mechanisms: (a) viral persistence and reactivation of latent viruses, including ancestral retroviruses (1), (b) subacute inflammation and autoimmune processes (1), (c) mitochondrial dysfunction (13), (d) increased intestinal permeability, microbial translocation, and dysregulation of the gut-brain axis (1), (e) endothelial senescence, including apoptosis, ferroptosis, and telomere shortening (14). These processes may run individually or in combination and can impair cellular metabolism, mitochondrial function, and neurotransmitter synthesis, leading to systemic dysregulation (13).

While Guillain-Barré syndrome (GBS) is not classified as a post-acute syndrome, it represents a related post-infectious condition with similar immunological and clinical features and was frequently investigated in the studies retrieved by our literature search. GBS is an acute, monophasic autoimmune disease of the peripheral nervous system with well-defined diagnostic criteria (1), distinguishing it from PAIS. Nevertheless, owing to its post-infectious onset, immune-mediated pathogenesis, and its frequency in our search results, we included GBS in this review to enable a mechanistic comparison to support biological interpretations. GBS will be briefly investigated in a dedicated section of this review to enable a focused comparison with PAIS entities (15).

Here, we present the results of a comprehensive systematic literature review on biomarkers associated with PAIS. Particular attention has been given to PACS and ME/CFS, which represent the most extensively studied PAIS entities to date. The high misdiagnosis rates, combined with the limited understanding of the pathogenesis of PAIS and the lack of targeted therapeutics and diagnostic tests, highlight the strong need to identify reliable biomarkers and potential pathomechanisms. This review provides a novel and comprehensive perspective on PAIS. Previous reviews have typically focused on a single condition, such as PACS or ME/CFS, and have often relied on a single omics approach, or, at most, limited multi-omics analyses*. Clarke* et al. summarized blood-based biomarkers in ME/CFS (13), whereas *Moreno-Corona* et al. analyzed microbiota dynamics in the context of PACS (16). In contrast, our work systematically covers the full spectrum of PAIS and integrates multiple omics layers, offering a holistic view of these syndromes across diverse molecular domains.

By systematically reviewing and synthesizing the available evidence, this work aims to highlight both the differences and the commonalities between the infectious agents and disease entities that lead to post-acute sequelae. Identifying shared biomarker patterns may provide insight into overarching mechanisms, whereas disease-specific signatures could inform tailored diagnostic strategies and therapeutic approaches. We first summarize and categorize the results, then integrate our findings in the discussion, proposing a disease framework that connects biomarkers and pathways to potential mechanisms of disease. In doing so, this review seeks to contribute to a deeper understanding of the biological basis of PAIS and related syndromes, and to outline future directions for biomarker-driven research.

## 2 Methods

This systematic review followed the 2020 Preferred Reporting Items for Systematic Reviews and Meta-Analyses (PRISMA) guidelines (17). A systematic search was conducted in PubMed and Web of Science, with the final search completed on April 16th, 2026. All available studies published up to this date were included in this review. The full search string is provided in **Table 1**, and the completed PRISMA checklist is available in the supplementary material [see **Additional File 1**].

**Table 1:** Search strategy for PubMed and Web of Science by topics corresponding to the inclusion criteria.

Conditions of interest AND	Analyzed biological molecules AND	Biological end points
(“Post-infectious Disorders”[Mesh] OR ((“post-acut*” OR “post-infect*”) AND (“sequelae*” OR “syndrom*” OR “disorder*”)) OR “long-covid” OR “post-covid” OR “Post-Acute COVID-19 Syndrome”[Mesh] OR “post-ebola*” OR “post-lym*” OR “Post-Lyme Disease Syndrome”[Mesh] OR “myalgic encephalomyelit*” OR “chronic fatigue syndrom*”)	(“epigenom*” OR “exom*” OR “genom*” OR “glycom*” OR “metabolom*” OR “microbiom*” OR “lipidom*” OR “multi-omic*” OR “multiomic*” OR “Multiomics”[Mesh] OR “omic*” OR “proteom*” OR “transcriptom*”)	(“biomarker*” OR “Biomarkers”[Mesh] OR “pathomechani*”)
Complete search term		
“Post-infectious Disorders”[Mesh] OR ((“post-acut*” OR “post-infect*”) AND (“sequelae*” OR “syndrom*” OR “disorder*”)) OR “long-covid” OR “post-covid” OR “Post-Acute COVID-19 Syndrome”[Mesh] OR “post-ebola*” OR “post-lym*” OR “Post-Lyme Disease Syndrome”[Mesh] OR “myalgic encephalomyelit*” OR “chronic fatigue syndrom*”) AND (“epigenom*” OR “exom*” OR “genom*” OR “glycom*” OR “metabolom*” OR “microbiom*” OR “lipidom*” OR “multi-omic*” OR “multiomic*” OR “Multiomics”[Mesh] OR “omic*” OR “proteom*” OR “transcriptom*”) AND (“biomarker*” OR “Biomarkers”[Mesh] OR “pathomechani*”)		

### 2.1 Research question

The research question was formulated using the PICOS framework (18). In humans with post-acute infection syndrome (PAIS) (P), how do biological and clinical parameters (I) (e.g., inflammatory markers, metabolic profiles, immune cell profiles) differ between individuals with PAIS and control groups, including healthy individuals, those in the acute phase of infection, fully recovered individuals, or patients with other chronic conditions (C) as assessed in controlled human studies (S). Outcomes of interest included immunological, metabolic, and clinical biomarkers (O).

### 2.2 Search strategy

The search included keywords related to different conditions of interest, analyzed biological molecules, and biological endpoints. The search strategy employed text and index terms, as well as synonyms and related terms for each keyword. The search also used Boolean operators ‘AND’ and ‘OR’ to combine the search terms, as well as truncation to include word variations [*]. The Boolean operator ‘AND’ is used to connect the three overarching topics: the condition of interest, the biological molecules analyzed, and the biological endpoints. The ‘OR’ operator is used to enumerate the more specific terms within a given topic. An overview of the full search strategy is shown in **Table 1**. The full PubMed search term is provided in **Table 1**. The search term was adapted for Web of Science by removing the search field tags. The inclusion and exclusion criteria were established before commencing any searches.

### 2.3 Inclusion and exclusion criteria

Full-text peer-reviewed original studies were eligible if they met the following inclusion criteria: (1) investigation of patients with PACS, ME/CFS, or other PAIS; (2) reporting of laboratory or omics biomarkers of PAIS; (3) aiming to identify potential diagnostic markers, therapeutic targets, or potential pathomechanisms of the syndromes. We excluded article types such as reviews, editorials, comments, opinions, letters, and study protocols, as well as articles that were not peer-reviewed or not available in English. Studies were further excluded if they focused on animal models, other aims (technological, methodological, bioinformatics, or epidemiological), or conditions outside PAIS. In addition, we excluded case reports and studies that did not include a comparison between PAIS and a control group (other PAIS, healthy, or recovered), or lacked data specifically on PAIS (i.e., including PAIS patients but focusing on comorbidities or predictive biomarkers before onset of disease), or did not report any biomarkers. An overview of the inclusion and exclusion criteria is given in **Table 2**.

**Table 2:** Inclusion and exclusion criteria.

Description	Included	Excluded
Type of article	Original peer-reviewed research studies written in English	Preprint Review Single-case Protocol Editorial Commentary Opinion Letter
Type of participants	Human	Animal model Plant model
Focus of study	Disease research	Technical Methodological Bioinformatical Epidemiological
Type of disease	Post-acute infection syndromes	Other conditions Guillain-Barré Syndrome COVID-19 without long-term effects
Outcome measures	Biomarker Omics Pathomechanisms	Only symptoms No biomarkers

GBS studies were separately included to enable mechanistic comparison with other post-infectious syndromes and to explore overlapping biomarker signatures.

### 2.4 Operational definition of PAIS

For the purposes of this review, PAIS was operationally defined as the presence of persistent, recurrent, or newly emerging symptoms, clinical abnormalities, or biological alterations occurring after the acute phase of an infectious disease and temporally separated from the initial infection episode. Inclusion was based on a temporal criterion, rather than on specific symptom patterns or etiological mechanisms. Conditions were classified as PAIS if long-term effects were reported beyond the acute illness period, regardless of the terminology used in the original study (e.g., post-COVID, long COVID, or PACS).

Post-COVID, long COVID, and PACS are all used to describe persistent symptoms and long-term complications following acute SARS-CoV-2 infection. Although these terms are often treated as distinct entities, their definitions vary substantially across published studies and authoritative sources. Different time thresholds, symptom constellations, and diagnostic criteria are applied, resulting in considerable heterogeneity in case definitions. Consequently, a strict conceptual separation of post-COVID, PACS, and long COVID is not feasible within the scope of this review, as doing so would conflict with the variable definitions employed in the included studies. For this reason, no single operational definition was imposed for any of these terms, and the term PACS was used throughout the manuscript to refer to all of them.

Accordingly, study inclusion under the umbrella of post-acute infection syndromes was primarily based on temporal classification, requiring the presence of long-term or persistent effects occurring after the acute phase of infection. All conditions characterized by symptoms or biological alterations emerging or persisting at a time point clearly separated from the initial illness episode were considered eligible.

### 2.5 Data extraction and synthesis

Two independent reviewers (KW and MS), both with biomedical expertise, screened all studies eligible for inclusion. Disagreements were resolved through discussion and consensus. From each included publication, key information was extracted, including author, publication year, patient demographics (age, sex, number of participants), case definitions of ME/CFS, PACS, or PAIS, type of case and control groups (e.g., healthy, recovered, other diseases), analytical methods, reported conclusions, sample type, country of the cohort, and study limitations. For the studies included, we first extracted all explicitly named analytes irrespective of their significance. We then identified the most important biomarker results (e.g., up- or downregulation, changes, unchanged), irrespective of the measurement time point or analytical method.

### 2.6 Statistical analysis, presentation and visualization

A descriptive analysis was conducted to compare subgroups (patients, healthy controls, PACS patients, and ME/CFS patients). Subgroup analyses by disease type and analytical method were performed to explore heterogeneity, with the results presented in a separate summary table (**Supplementary Table 3**). The results are presented in overview tables complemented by graphical displays.

For the analysis of patient demographics, only cohorts reporting the mean age of participants were included. From these cohorts, the overall mean age and median age were calculated. In studies reporting more than one cohort, each cohort was analyzed separately. The same procedure was applied specifically for cohorts of patients with ME/CFS and, separately, for those with PACS.

The sex distribution was analyzed analogously. For each cohort in which the sex distribution was reported, the proportion of male participants was extracted or calculated. Based on these values, the overall mean, standard deviation, and median were calculated across all patient and control cohorts. Separate analyses were also performed for the ME/CFS and PACS cohorts.

Violin plots were used to illustrate the distributions of age, sex, and symptoms, whilst bar charts were used to visualize the participant numbers, cohort countries, publication years, and the distribution of omics methods across disease groups. Pie charts were used to show the proportions of PAIS subtypes (e.g., PACS, ME/CFS). The main findings, particularly frequently reported molecules and patterns, were integrated into the results section, whereas individual molecules or single-study findings were not described in detail in the main text.

Given the heterogeneity among the included cohorts and the nonnormality of the demographic data, nonparametric tests were used. Group comparisons were performed via the Mann-Whitney U test. Analyses were conducted both between the patient and control cohorts and between the ME/CFS cohorts and PACS cohorts. Comparisons included both age and sex distributions. For each test, p-values, z-scores, and effect sizes (r) were calculated to evaluate the magnitude and statistical significance of group differences.

### 2.7 Sensitivity analyses, certainty of evidence, and reporting bias

The robustness of the findings was examined through sensitivity analyses that accounted for sample sizes in each study. Study design, sample characteristics, and level of evidence were systematically assessed using modified Grading of Recommendations Assessment, Development, and Evaluation (GRADE) (19) and summarized in tabular form to enable a structured evaluation of heterogeneity and to support the interpretation of conflicting findings in the discussion. For this purpose, an initial score for uncertainty of evidence was assigned based on study design, with randomized controlled trials receiving the best starting score of 1, followed by prospective case-control/cohort studies, retrospective case-control/cohort studies, cross-sectional studies, and pilot studies. The initial score was subsequently adjusted by applying penalties for imprecision (based on sample size), indirectness, and risk of bias. Inconsistencies across studies were systematically documented but were not incorporated into the numerical penalty system. The final score ranged from 1 to 11 points, with 1 being the best possible score, and was categorized as low (1–4), moderate (5–7), or high (8–11) uncertainty of evidence. In addition, study limitations were extracted and documented to inform the assessment of the certainty of evidence. To further address inconsistent and contradictory findings across studies, a systematic analysis of potential sources of heterogeneity was conducted. Reporting bias was minimized by including the main findings of each study in the summary tables. Given that the majority of included studies were case-control and cohort studies, the quality of non-randomized studies was additionally assessed using the Newcastle-Ottawa Scale (NOS) to allow a more detailed methodological evaluation (20). To facilitate interpretation and transparency, the quality assessment results were additionally visualized as a heatmap and bar plots. The quality assessment is performed using a star-based scoring system, in which studies are awarded stars for predefined criteria across the three domains, enabling evaluation of methodological quality and potential risk of bias, with higher scores indicating higher study quality. The NOS evaluates eight items categorized into three groups: the selection of exposure and nonexposure groups; the comparability of these groups; and the comparability based on the study design (which provides an additional star). Therefore, the outcome evaluation may reach a maximum of nine stars. A total of seven or more stars implies a low risk of bias, while five to six stars are considered moderate risk, and zero to four stars are considered high risk.

## 3 Results

### 3.1 Study selection

**Figure 1:**
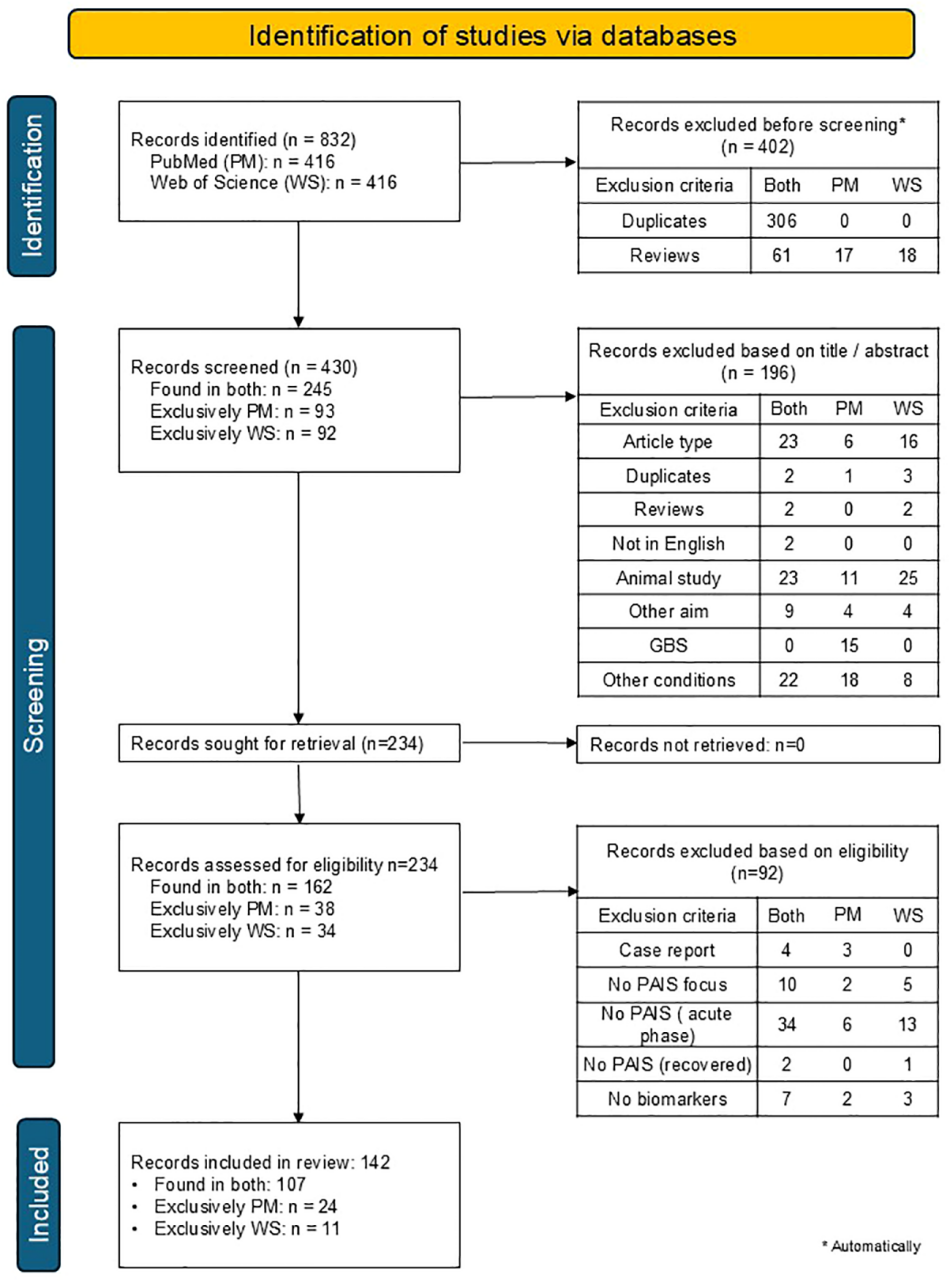
The PRISMA flow diagram illustrates the study selection process. The diagram shows the identification, screening, eligibility assessment, and inclusion of studies in the systematic review. The numbers indicate the records at each stage, including the reasons for exclusions.

The literature search yielded 430 results after removing duplicates. After screening the titles and abstracts, 234 full texts were assessed for eligibility, with 142 studies meeting the inclusion criteria (21–162) (**Figure 1**).

### 3.2 Study characteristics

The included publications describe analyses of patients with PACS (including post-COVID-19 syndrome and long-COVID), ME/CFS, acute neuroborreliosis, and posttreatment Lyme disease syndrome (PTLDS). 87 publications addressed PACS (61.3%), and another 48 publications ME/CFS (33.8%). Five publications (3.5%) analyzed the combination of ME/CFS and PACS, and only one paper investigated PTLDS and acute neuroborreliosis (0.7%).

Various omics-based or biomarker-based methods have been used to analyze datasets. Proteomics was the most frequently used approach (43.4%), followed by metabolomics (18.2%), genomics (11.9%), and transcriptomics (9.8%). The microbiome (9.1%), non-omics biomarkers (4.2%), and multi-omics (3.5%) were less common.

The most frequently used methods for the analysis of PAIS have been mass spectrometry (25 studies) and ELISA (15 studies). Olink proteomics panels, flow cytometry gene profiling, and NMR-based metabolomics had been utilized in eight studies. In addition, machine learning tools (5 studies), such as large language models and classification algorithms, and Western blotting, which had been reported in 4 studies, were applied.

**Figure 2:**
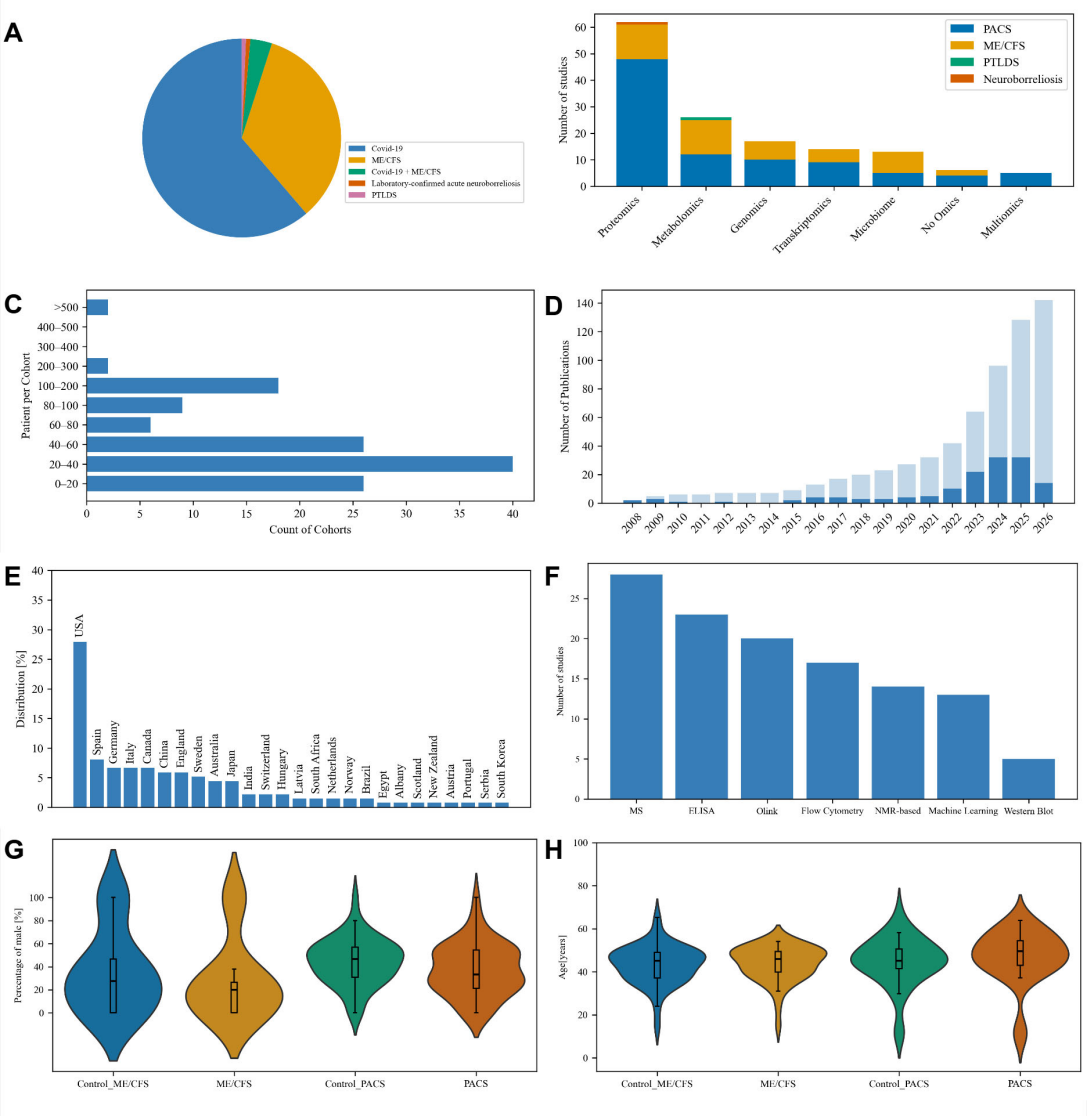
Overview of the studies included. **(A)** Pie chart showing the proportional distributions of different PAIS. **(B)** Bar chart of omics approaches stratified by PAIS (PACS, ME/CFS, PTLDS, Lyme disease). **(C)** Bar plot showing the distribution of cohorts across patient number bins, from 0–20 to over 500 participants. **(D)** Bar plot showing the number of publications per year, presented as absolute and relative values. **(E)** Bar plot depicting the proportional distribution of study cohorts by country/ethnicity. **(F)** Bar plot showing the distribution of the most frequently used methods. **(G)** Violin plot comparing the sex distribution between patients and controls. **(H)** Violin plot comparing the age of ME/CFS and PACS cohorts with their respective controls.

There has been a marked increase in publications since 2022 for both ME/CFS and PACS. While only 32 studies were published between 2008 and 2021 (~2.5 studies/year), 10 appeared in 2022 alone, followed by an additional 100 publications from 2023 onward (28/year) (**Figure 2D**).

### 3.3 Patient characteristics

The total number of patients analyzed was 8854. On average, the number of patients per study was 66.6 ± 131.9 (range 3-1,194), with a median of 39. The average age in the control group for ME/CFS was 43.1 ± 9.1 years, and that in the control group for PACS was 44.4 ± 11.7 years, with a median of 44.7 years in ME/CFS control group and 45.0 years in PACS controls. The age ranges of the different cohorts differed greatly (**Figure 2H**). Some studies focused on older age groups, e.g., 53–73 years (21), whereas others included patients of various ages, e.g., 3–84 years (22). The average age of PACS patients was (47.1 ± 12.3 years), and for ME/CFS patients (44.0 ± 8.1 years). A statistically significant age difference was observed between the PACS and ME/CFS groups (p = 0.036), with ME/CFS patients being younger than PACS patients (Z = −2.11). The effect size was moderate and therefore clinically relevant (r = 0.230). In contrast, no significant age differences were found between the PACS and control groups, or between the ME/CFS and control groups (p > 0.05 for both comparisons).

The sex distribution was inhomogeneous. Some publications analyzed only male or female patients, whereas others used various sex distributions. The average sex distribution among the control group for ME/CFS was at 35.4% ± 34.9 male (median 27.5% male) and 44.7% ± 20.3 male (median 46.7% male) among the control group for PACS (**Figure 2G**), with 37.5% ± 22.7 male in the PACS group *vs* 28.9% ± 33.9% male in the ME/CFS group. A statistically significant difference was observed between the ME/CFS and PACS groups (p = 0.031), with ME/CFS patients showing a lower proportion of male participants compared to PACS patients (Z = −2.16). The effect size was small to moderate (r = −0.267), indicating a limited but statistically relevant difference between groups. In contrast, no significant sex differences were found between the PACS and control groups, or between the ME/CFS and control groups (p > 0.05 for both comparisons).

The geographical distribution was unbalanced. The majority of participants in the reviewed cohorts were from North America (35.3%) or Europe (45.6%). The Asian cohort accounted for 13.2%, the Australian cohort for 5.2%, the African cohort for 2.2%, and South America for 1.5%. Participants from Antarctica were not included in any cohort (**Figure 2E**). The included studies revealed differences in the distribution of ethnicities investigated between the ME/CFS and PACS cohorts. While ME/CFS studies covered fewer countries and were concentrated more in specific regions, PACS publications spanned a broader range of countries. Notably, North America, particularly the United States, was represented in the ME/CFS cohorts (n = 16) just as frequently as in the PACS cohorts (n = 14). In contrast, European countries were more commonly included in PACS studies. Countries such as Switzerland, Hungary, Australia, and Estonia appeared exclusively in PACS publications, but not in ME/CFS studies. The ethnic and geographical compositions of the study cohorts differed between the two disease groups.

With respect to the study setting, 18 different test-control group combinations were observed. Forty-nine studies (34.5%) compared ME/CFS to healthy controls, 25 studies (17.6%) compared PACS to healthy controls, and 24 studies (16.9%) compared PACS to acute or recovered COVID-19 patients (**Supplementary Table 3**). In the comparison of PACS versus healthy groups, the analyses focused on genomics, proteomics, transcriptomics, microbiome, and metabolomics data. A variety of analytical methods, including flow cytometry, ELISA, mass spectrometry, and Western blotting, have been applied to these data. Similarly, in the PACS versus COVID-19 comparison and the ME/CFS versus healthy comparison, these same molecular layers were investigated. Even when the same type of omics was analyzed, different methods were applied within each group, increasing heterogeneity. At the molecular level, direct comparisons between studies are not feasible. For example, protein analyses often rely on panels that measure only a predefined set of molecules. Similarly, ELISA, Western blotting, and both flow cytometry and custom assays target specific molecules, allowing conclusions only about the presence or absence of changes in those selected targets, rather than providing a comprehensive overview of all proteins or molecules in a sample. As a result, comparisons were performed at the pathway level, enabling the integration of findings across studies despite methodological differences and targeted focus.

Among the included cohorts, blood samples were the most frequently analyzed, used in 113 cohorts (79.6%). Fecal samples were examined in sixteen cohorts, urine in five cohorts, and cerebrospinal fluid (CSF) in three cohorts. Lung tissue, bronchoalveolar lavage fluid (BALF), exhaled breath condensate (EBC), and circulating cell-free mitochondrial DNA (ccf-mtDNA) were each assessed in a single cohort.

### 3.4 Risk of bias

As shown in the evidence table (**Supplementary Table 1**), the majority of the included studies were prospective cohort studies (n = 103), followed by retrospective cohort studies (n = 25), and cross-sectional studies (n = 7). In addition, seven studies were classified as pilot studies, while one study was categorized as a randomized controlled trial. Accordingly, the initial uncertainty of evidence score (I.S.) was assigned based on study design, with randomized controlled trials receiving the best score of 1, prospective cohort studies a score of 2, retrospective cohort studies a score of 3, cross-sectional studies a score of 4, and pilot studies the worst score of 5.

**Figure 3:**
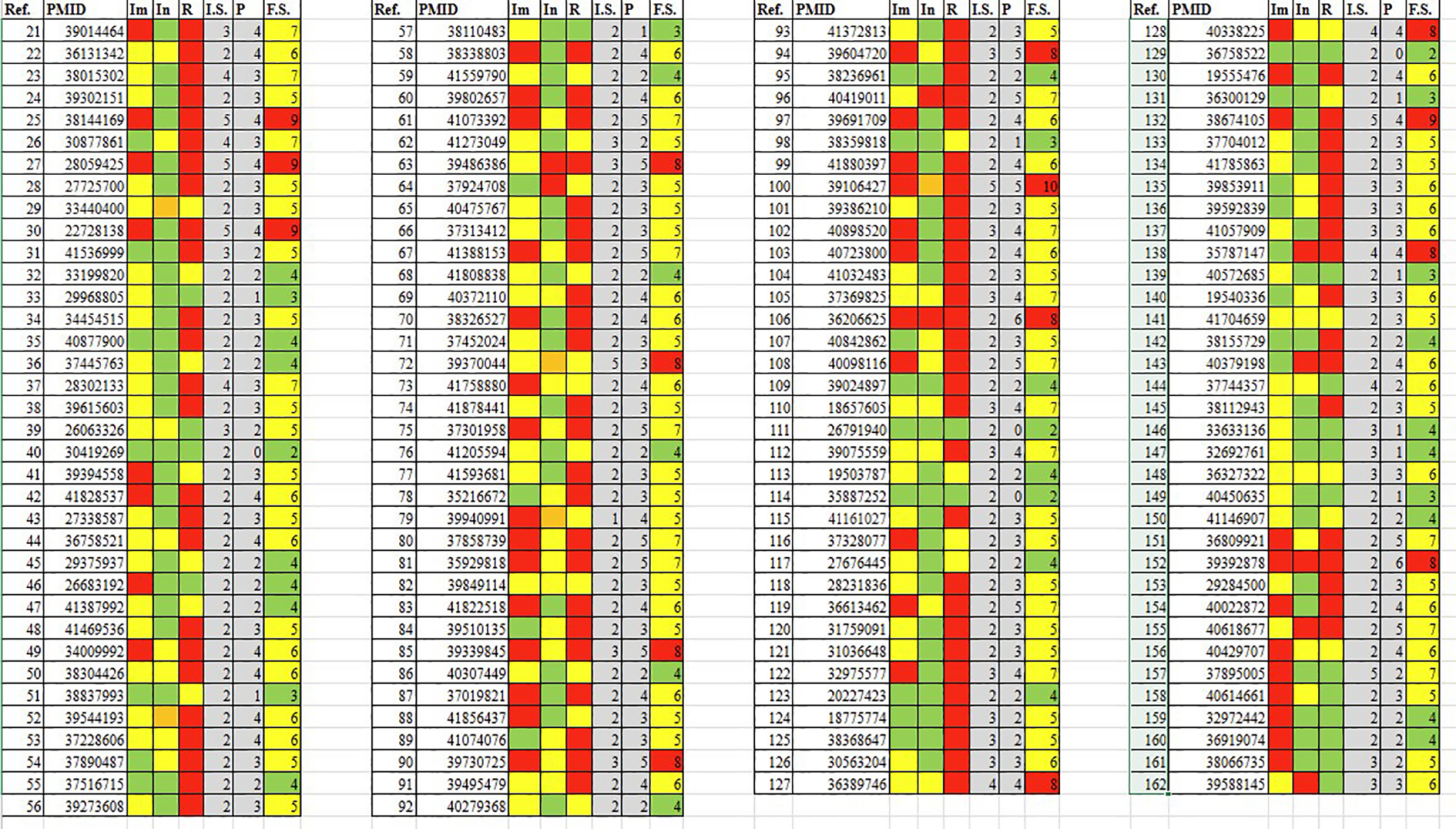
Heatmap visualization of the modified GRADE assessment. The heatmap summarizes the modified GRADE-based evidence assessment for the included studies. Ref. indicates the study reference. Im refers to imprecision, which was restricted to sample size; In indicates inconsistencies across studies; I.S. represents the initial score assigned according to study design; P indicates applied penalties; and F.S. represents the final score. Color coding was applied to Im and F.S., with red indicating high concern, yellow/orange indicating moderate concern, and green indicating low concern.

The final evidence score was determined by applying penalties for imprecision, indirectness, and risk of bias. Inconsistencies across studies were documented separately to support the interpretation of conflicting findings, but were not incorporated into the numerical scoring system. Based on the final score, 34 studies (23.9%) were classified as having low uncertainty in the evidence, 93 studies (65.5%) as having moderate uncertainty, and 15 studies (10.6%) as having high uncertainty (**Figure 3**).

Among the individual penalty domains, indirectness was generally low, with only nine studies receiving a high-risk rating. In contrast, imprecision, assessed primarily based on sample size, represented a more substantial limitation, with 48 studies being classified as having a high risk of imprecision. These findings indicate that limitations in sample size rather than indirectness were the predominant factor reducing overall evidence certainty across the included studies.

**Figure 4:**
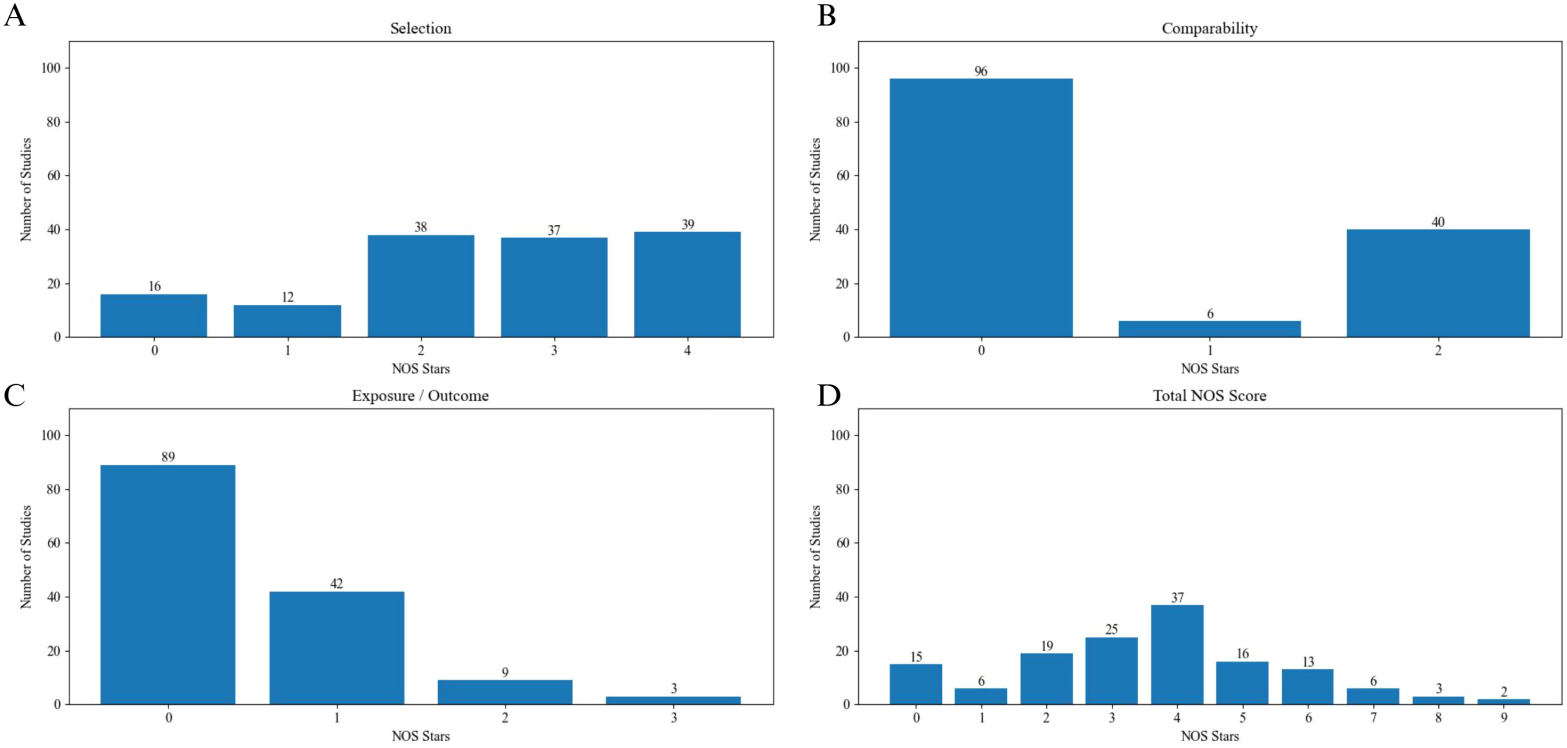
Distribution of Newcastle-Ottawa Scale (NOS) scores among included studies. Bar charts showing the distribution of awarded stars across included studies according to the Newcastle-Ottawa Scale (NOS) domains: **(A)** Selection, **(B)** Comparability, **(C)** Exposure/Outcome, and **(D)** total NOS score. The x-axis represents the number of stars awarded, while the y-axis indicates the number of studies.

**Figure 5:**
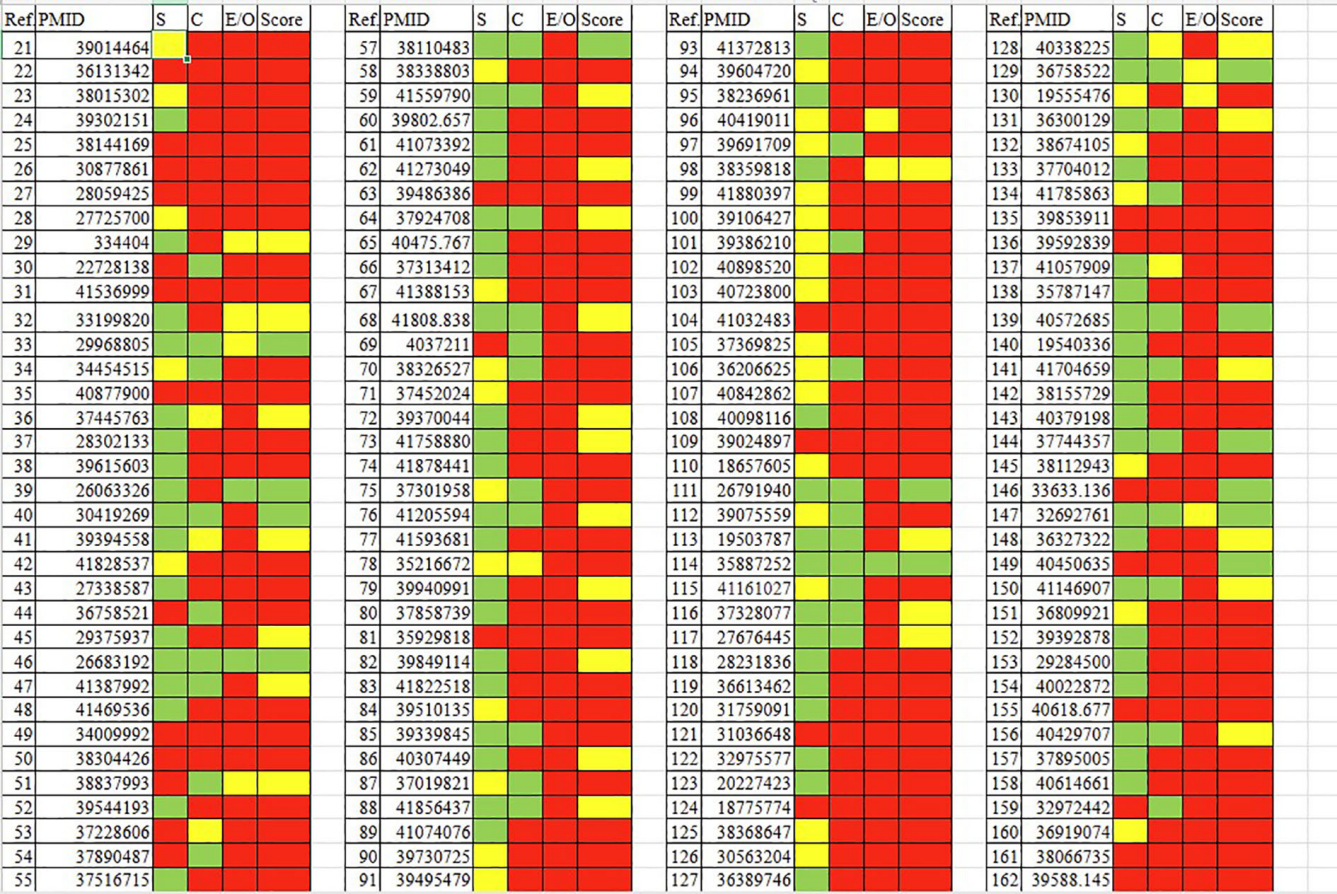
Newcastle-Ottawa Scale (NOS)-based risk of bias assessment across included studies. The table summarizes the methodological quality assessment of included studies using the Newcastle-Ottawa Scale (NOS). Columns include study reference (Ref), PubMed ID (PMID), Selection (S), Comparability (C), Exposure/Outcome (E/O), and the overall NOS score. Risk of bias was visually represented using a color-coded scheme. For the Selection domain, scores of 0–1 were classified as high risk of bias (red), 2 as moderate risk (yellow), and 3–4 as low risk (green). For Comparability, scores of 0 indicated high risk, 1 indicated moderate risk, and 2 indicated low risk. For Exposure/Outcome, scores of 0–1 were classified as high risk, 2 as moderate risk, and 3 as low risk. The overall NOS score was categorized as high risk (0-4, red), moderate risk (5-6, yellow), and low risk of bias (7-9, green).

To enable a more detailed assessment of the methodological quality of the studies and their potential risk of bias, the Newcastle-Ottawa Scale (NOS) was applied to the case-control and cohort studies. The NOS assessment revealed that most studies were classified as having a high risk of bias (n = 102), followed by a moderate risk (n = 29). Only a limited number of studies were considered to have a low risk of bias (n = 11). The detailed results are shown in **Supplementary Table 1**; **Figure 4** and **5**.

A domain-specific evaluation revealed significant variations in the quality of methodological reporting. Within the participant selection domain, more methodological information was available on average, enabling higher NOS ratings. However, 11.3% of studies still received no stars due to missing or inadequate reporting, or the absence of a common/standardized procedure. By contrast, substantially larger proportions of studies received no stars in the comparability of participants (67.6%), due to a lack of age- and sex-matched controls. And exposure/outcome assessment domains (62.7%), due to a lack of outcome validation, the same control and test procedure, and reporting dealing with missing values, indicating important methodological limitations and inadequate reporting (**Supplementary Table 1**). Overall, 10.6% of studies received no NOS stars, further highlighting the heterogeneity in study quality (**Figure 4**).

As illustrated in **Figure 5**, the color-coded heatmap indicates that the risk of bias was generally lower in the selection domain compared with the comparability and exposure/outcome domains. While many studies achieved comparatively favorable ratings for selection, substantially higher risks of bias were observed for comparability and exposure/outcome assessment. Overall, the total risk of bias was frequently classified as high, whereas only a limited number of studies demonstrated a low or moderate overall risk of bias (**Figure 5**).

### 3.5 ME/CFS

This review includes fifty-three studies investigating ME/CFS via a range of omics approaches, including metabolomics, proteomics, transcriptomics, and microbiome analyses (**Supplementary Table 2**). In these studies, consistent patterns of metabolic dysregulation, immune dysfunction, and impaired energy homeostasis were reported.

#### 3.5.1 Amino-acid metabolism

**Figure 6:**
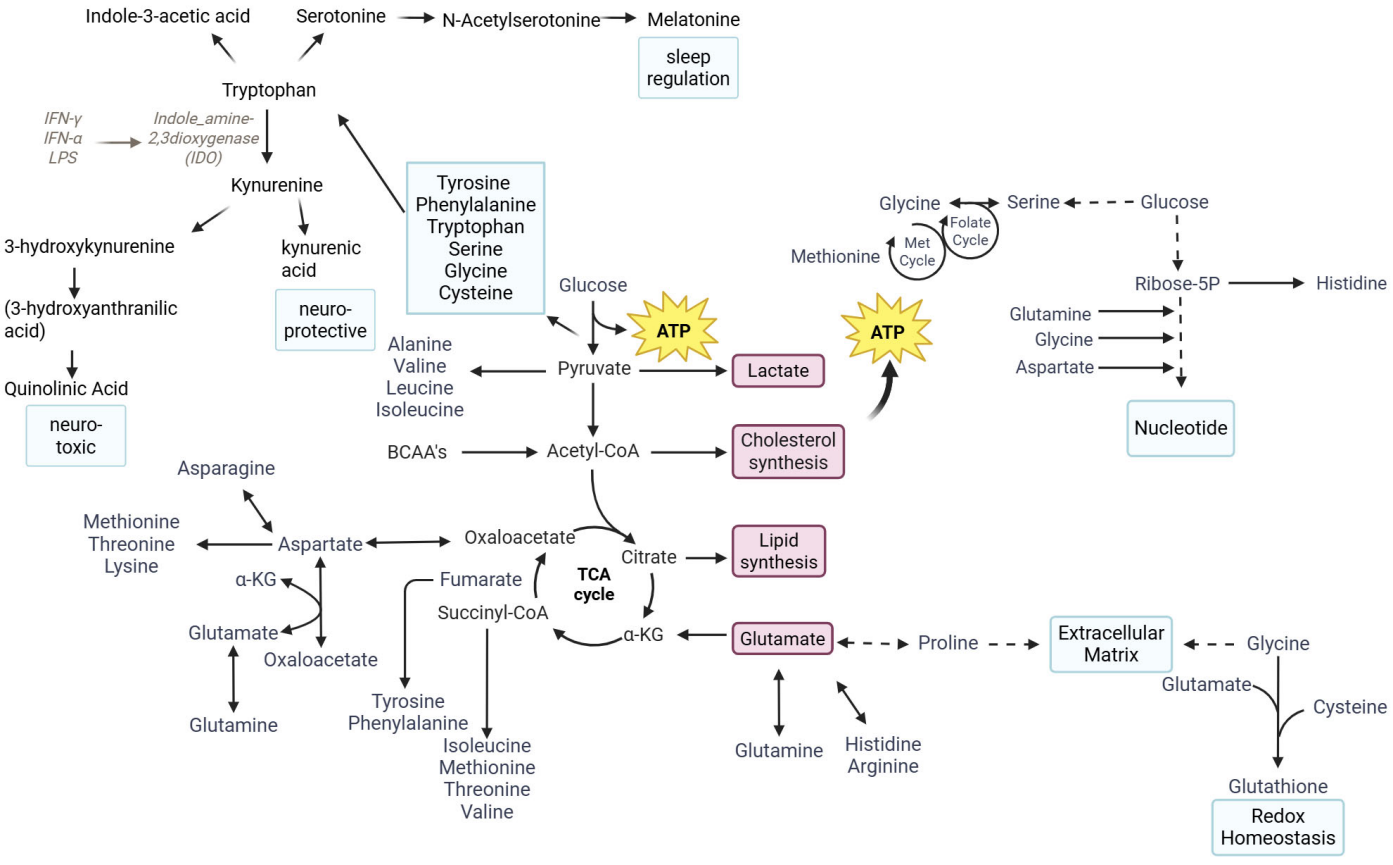
Alterations in the amino acid pathway in PAIS patients. The diagram illustrates key molecules and their interconversions within the amino acid metabolic pathway. Many of these metabolites are altered in PAIS patients, indicating a disruption of normal amino acid metabolism. Created in BioRender. Wendt, K. (2025) https://BioRender.com/hlcp87y.

A recurrent finding was the dysregulation of multiple metabolic pathways, particularly those affecting amino acid, lipid (**Figure 6**), and energy metabolism. Alterations in the tryptophan-kynurenine pathway (TKP), characterized by a disrupted tryptophan-kynurenine ratio (TKR), reduced NAD^+^ levels, or reduced quinolinic acid (QUIN) concentrations, have been reported in several studies. A reduction in 3-hydroxykynurenine (3-HK), quinolinic acid, 3-hydroxyanthranilic acid (23, 24), and serotonin (24) was observed. *Taenzer* et al. demonstrated dysregulation of the TKR (25), in which reductions in the levels of 3-hydroxykynuene, 3-hydroxyanthranilic acid, indole-3-acetic acid, serotonin, and anthranilic acidshift the TKP towards KYNA, as the other three pathways are reduced (**Figure 6**).

In addition to alterations in the TKP, several other amino acid metabolism pathways were also affected in ME/CFS, including those involving phenylalanine, tyrosine, glutamine, glutamate, branched-chain amino acids, histidine, and methionine. Two studies reported alterations in the phenylalanine-tyrosine pathway (24, 25). Furthermore, phenylalanine was reduced in ME/CFS (24, 25). In addition to the reported decrease in phenylalanine*, Zhang* et al. reported altered tyrosine levels (26). Five studies reported findings related to glutamate and/or glutamine (26–30), where the glutamine-glutamate metabolism was affected. *Armstrong* et al. reported reduced glutamine levels (30), whereas *Baraniuk* et al. reported elevated glutamate levels (29), while glutamate levels were reduced (27) in *Germain* et al. Differences in study design and sample type may explain the observed discrepancy. *Baraniuk* et al., being a prospective study comparing patients and controls (29), provides more robust evidence and a higher NOS Score (NOS: 6 stars, GRADE: 7) than *Germain* et al. (NOS: 0 stars, GRADE: 9), which is limited by its pilot study design (27). Additionally, the use of different biological samples (blood (27) *vs*. CSF in (29)) may further contribute to the divergent findings (**Supplementary Table 4**). *Zhang* et al. identified alterations in both glutamine and glutamate (26), whereas *Yamano* et al. reported no significant changes in ME/CFS (28). The findings differed between studies, with *Yamano* et al. (28) employing a prospective design and comparing ME/CFS patients with healthy controls. In contrast, Zhang et al. (26) used a cross-sectional approach and compared CFS patients with individuals with depression. However, both studies have moderate certainty of evidence and show a high risk of bias, with a NOS score of 3 and a GRADE rating of 5 in Yamano et al., and a NOS score of 0 and a GRADE rating of 7 in *Zhang* et al. Additionally, different biological samples were analyzed (blood only *vs*. blood and urine), which may contribute to the observed variation in results (**Supplementary Table 4**). Three studies reported alterations in urea cycle metabolites. On the one hand, ornithine levels were reduced (30), whereas on the other hand, they were increased and accompanied by decreased urea and citrulline levels (28). Again, the findings of *Yamano* et al. indicating increased leucine levels can be considered more robust, as they are based on a prospective study design. In contrast, the results reported by *Armstrong* et al. are limited by the exploratory nature of a pilot study (30). Regarding the risk of bias, both studies indicate a high risk, with a rating of three stars (28, 30) and moderate to high uncertainty (GRADE: 5 and 9). The third study showed changes in alanine aminotransferase, alkaline phosphatase, bilirubin, uric acid, and urea (31). Similarly, contradictory findings have been reported for branched-chain amino acids (BCAAs). *Zhang* et al. reported a change in leucine (26), but *Yamano* et al. reported no alterations in valine, leucine, or isoleucine (28), whereas reduced levels of valine and isoleucine were detected in *Germain* et al. (27). Altered levels of histidine were reported by both *Zhang* et al. and *Germain* et al. (26, 27). Changes in methionine metabolism were also observed, with decreased levels of adenosylhomocysteine (24) and methionine (27).

Overall, reduced phenylalanine levels reduce the substrates available for tyrosine synthesis, resulting in deficiencies of key neurotransmitters. Reduced ornithine levels disrupt the urea cycle, leading to decreased urea production and secondary decreases in citrulline and homocysteine. Reduced levels of valine and isoleucine (both branched-chain amino acids) decrease energy production via the TCA cycle, as these amino acids are necessary for the formation of acetyl-CoA and succinyl-CoA. Conversely, an increase in these amino acids enhances TCA cycle activation and increases energy production. Glutamate and glutamine are in a delicate metabolic balance: a reduction in glutamate leads to reduced glutamine synthesis, impairing ammonia detoxification and further destabilizing the TCA cycle via the glutamine degradation pathways (**Figure 5**). Overall, changes in amino acid, metabolite, and enzyme concentrations disrupt metabolic homeostasis, which can manifest clinically as organ dysfunction, particularly in the liver and kidneys.

#### 3.5.2 Energy metabolism

Energy metabolism impairments have been reported, including decreased ATP production and altered glycolysis (27, 32). An increase in pyruvate levels, indicating altered glycolysis (28), may suggest a possible redirection of energy towards glycolysis, similar to observations in cancer and chronic neuropsychiatric or cardiovascular diseases. Alterations in creatine (32) and creatine kinase levels (32) further indicate disruptions of the creatine-phosphate energy system.

#### 3.5.3 Lipid metabolism

Lipid metabolism alterations, along with significant changes in immune-modulatory lipids, were observed with notable sex-specific patterns. Altered sphingolipid levels were observed in two studies: both reported increased ceramide levels (33, 34), but one reported decreased sphingomyelin levels (33) and the other, in contrast, increased sphingomyelin levels (33). *Nagy-Szakal* et al. have a low risk of bias and good certainty of evidence (NOS: 8 stars, GRADE: 3) (33), whereas *Nkiliza* et al. have a moderate risk of bias and certainty of evidence (NOS: 4 stars, GRADE: 5) (34). Hexosylceramides showed sex-specific differences, being reduced in women but elevated in men, and phosphatidylinositol was also elevated in males (34). Changes in phospholipids and phosphatidylethanolamine (PE) were also sex specific, with lower levels in women and higher levels in men (34), whereas lysophosphatidylcholine (LPC) and phosphatidylcholine (PC) were decreased in men and increased in women (35). Triglycerides were generally elevated across cohorts (34, 35), with a pronounced increase in men (34). Elevated in men as well were omega-6 fatty acids, further supporting sex-specific lipid metabolic changes in ME/CFS (34). In addition, there were changes in apolipoproteins, which play an essential role in lipid metabolism, including increases in APO A-I and decreases in APOE (36).

Overall, an increase in triglycerides leads to an increase in ceramides, which reduces sphingomyelin levels. As hexosylceramides are derived from sphingolipids (including sphingomyelin), a reduction in sphingomyelin results in fewer hexosylceramides being produced. Omega-3 fatty acids exert negative feedback on LPC, which is why an increase in omega-3 leads to a reduction in LPC. PC and LPC are positively feedback-coupled, while LPC and apolipoproteins are negatively feedback-coupled. Therefore, a change in any of these molecules disrupts metabolic balance and leads to a disturbance in homeostasis.

#### 3.5.4 Inflammation pathways

Immune-related findings included dysregulation of the nuclear factor kappa-light-chain-enhancer of activated B cells (NF-κB pathway). With respect to inflammatory markers, *Lidbury* et al. observed no significant differences in IL-6, TNF-α, and IFN-γ (37). In contrast, other studies reported decreased levels of IL-6 (36) and TNF-α (36, 38), as well as general alterations (39, 40). The alterations are supported by evidence from prospective case-control studies, while *Lidbury* et al. used a cross-sectional design (37). Additionally, discrepancies may arise from differences in biological samples, as *Lidbury* et al. analyzed both blood and urine, whereas the other studies were limited to blood samples (**Supplementary Table 4**). With a NOS score of 7 and GRADE rating of 5 and 2 respectively, *Bhattacharjee* et al. and *Nguyen* et al. have a low risk of bias and moderate to good certainty of evidence (39, 40) compared to good certainty of evidence (GRADE: 4) but moderate risk of bias with a score of 6 by *Soffritti* et al. and moderate certainty (GRADE: 5 and 7) and high risks in the other studies with NOS scores of 3 (38) and 4 (37). Elevated levels of the inflammatory markers IL-5 and IL-8 have also been reported, indicating the activation of proinflammatory pathways (23). While one study reported a decrease in naïve CD4 cells (41), another study reported an increase in total T cells and CD4+ naïve cells, and decreases in NKs, monocytes, CD4+ TEM cells, and CD8+ naïve cells (42). *Sun* et al., with a rating of 6 stars (41), have a moderate, and therefore lower, risk of bias than *Seifert* et al., with a rating of 2 stars (42) and therefore a high risk. The certainty of evidence is comparable for both, with moderate ratings of 5 (41) and 6 (42).

#### 3.5.5 Epigenetic

Additionally, epigenetic modifications, including differential DNA methylation patterns, suggest potential epigenetic dysregulation in ME/CFS. MicroRNA profiling revealed increases in miR-127-3p, miR-142-5p, miR-143-3p, miR-150-5p, and miR-448 and decreases in miR-140-5p, reflecting the modulation of immune, inflammatory, and metabolic pathways (36). Further reductions were detected in the miRNAs hsalet-7b-5p and has-miR-374a-5p (43).

#### 3.5.6 Microbiome

Microbiome analyses revealed gut dysbiosis, including reduced β-diversity and decreased α-diversity, with reduced abundance of Firmicutes, which may contribute to microbial translocation and systemic inflammation (44–47). In *Cheng* et al., altered β-diversity and reduced α-diversity were observed (47), with reduced levels of Firmicutes (44, 47), Verrucomicrobiae, Clostridiales, Rikenellaceae, and Ruminococcaceae (47). In contrast, elderly patients showed an increase in alpha diversity (48). With a NOS score of 5, *Cheng* et al. have the lowest, but generally moderate risk of bias and good certainty of evidence (GRADE: 4), compared to a high risk with a NOS score of 3 and moderate certainty of evidence with GRADE ratings of 6 and 5, respectively (44, 48).

### 3.6 PACS

This review includes ninety-two studies investigating PACS via diverse omics approaches (**Supplementary Table 2**). Immune dysregulation, mitochondrial dysfunction, metabolic imbalance, and metabolic shift have emerged as central and recurrent findings across these studies.

#### 3.6.1 Amino acid metabolism

Amino acid metabolism exhibited extensive alterations in relation to PACS and showed sex-specific differences. TKR (25, 49) and kynurenine levels were increased (50), and, correspondingly, changes in TKR were associated with decreased tryptophan (51, 52). Additionally, a reduction in quinolinic acid was reported in blood (51), whereas *Taenzer* et al. reported increased levels of both quinolinic acid and kynurenine 3-monooxygenase (KMO) in urine (25). With a NOS score of 1 and a GRADE rating of 9, *Taenzer* et al. have a significantly higher risk of bias and greater uncertainty of evidence than *Khoramjoo* et al. with a NOS score of 5 and a GRADE rating of 3.

Tryptophan is converted into kynurenine, which is then broken down into QUIN in several steps via KMO (kynurenine 3-monooxygenase) (**Figure 6**). Considering all findings, an increase in QUIN shifts the kynurenine pathway towards a more neurotoxic state. Conversely, a smaller increase in quinolinic acid favors diversion into the kynurenic acid pathway, resulting in the formation of kynurenic acid, a neuroprotective metabolite. The balance between these two degradation pathways is crucial for neurological homeostasis.

Alterations in the glutamine-glutamate pathway were also observed. *Berezhnoy* et al. reported altered glutamate levels (53), *Holmes* et al. reported a reduced glutamate/glutamine ratio (49), and *Wang* et al. reported decreased glutamine levels (54). Furthermore, phenylalanine and tyrosine levels were both reduced, indicating broader disruptions in aromatic amino acid metabolism (53). Changes in the urea cycle included an increase in ornithine (54) and a decrease in arginine (51, 53).

As previously described in relation to ME/CFS, a reduction in glutamine leads to a reduction in glutamate, and a reduction in phenylalanine leads to a reduction in tyrosine. An increase in ornithine causes an increase in citrulline, which in turn leads to an increase in arginine and fumarate (**Figure 6**). An increase in ornithine combined with a decrease in arginine suggests a disruption of the urea cycle.

Branched-chain amino acids (BCAAs) and related amino acids exhibited notable alterations. Histidine showed inconsistent patterns across studies: one study reported decreased levels (50), while another reported increased histidine alongside reduced valine levels (53). The observed difference appears to be sex-related, as both the PACS and control groups consisted of comparable COVID-19 recovered or acute COVID-19 cohorts and similar blood-derived samples. However, *Saito* et al. included exclusively female participants (100% female) (50). In contrast, Berezhnoy et al. also included male participants in their samples (53) and had a slightly better NOS score (NOS: 2 stars, although both studies were classified as high risk and moderate certainty of evidence (both GRADE: 6). In males, however, leucine, isoleucine, and valine were increased, further highlighting sex-specific metabolic changes (55). General alterations in cysteine levels were observed (50), with changes in the cysteine pathway driven by elevated glycine occurring only in females (55). Methionine levels were also altered (50, 52), but were also reported to be increased in females (55).

In summary, during glycolysis, glucose is converted into histidine via ribose. Histidine and arginine can be converted into and from glutamate. Isoleucine, leucine, and valine are produced from succinyl-CoA (**Figure 6**). An increase in these BCAAs, therefore, indicates an increase in the TCA cycle.

#### 3.6.2 Energy metabolism

Energy metabolism was also affected, showing alterations in glycolysis, particularly in pyruvate and lactate levels. *Santana De Anda* et al. reported increased pyruvate levels (PACS *vs*. recovered group) (56). *Paris* et al. (PACS *vs*. healthy controls) reported an increase in lactate (57). In contrast, *Berezhnoy* et al. reported a decrease in both pyruvate and lactate levels (PACS *vs*. acute and recovered COVID-19 patients) (53), highlighting inconsistencies across studies. Different methods have been applied across these studies. *Berezhnoy* et al. and *Santana De Anda* et al. used flow cytometry with different panels (53, 56), whereas *Paris* et al. employed NMR-based metabolomic analysis (57), adding to the methodological diversity. The risk of bias and uncertainty was lowest for *Paris* et al. (57), with an NOS score of 7 and a good GRADE rating of 3, compared with NOS scores of 2 and GRADE ratings of 6 and 5 for the other two studies (53, 56). In addition, changes in choline, succinic acid, acetone, and glucose were observed (58).

The citrate acid cycle was also altered, with an increase in citrate (53) and malate (54). Changes in the creatine phosphate system were observed, as indicated by a decrease in ATP (50) and reductions in creatinine in males and females (53, 59) and urinary creatine in females. In contrast, serum creatine levels were increased in females (55). Glucose reacts to form pyruvate, releasing ATP. Pyruvate then reacts to form lactate, acetyl-CoA, and branched-chain amino acids (BCAAs). Acetyl-CoA enters the TCA cycle and reacts to form citrate, which is subsequently converted to malate (**Figure 6**).

Generally, an increase in lactate leads to a shift in pyruvate utilization towards lactate production, which reduces the availability of pyruvate for the formation of acetyl-CoA and branched-chain amino acids (BCAAs). This affects energy production in the TCA cycle and can lead to a reduction in citrate, malate, and ATP.

#### 3.6.3 Lipid metabolism

Lipid metabolism exhibited alterations, with one contradictory finding. *Wei* et al. reported reduced fatty acid levels in blood (60), whereas *Paris* et al. reported increased fatty acid levels in exhaled breath condensate (57). The risk of bias and uncertainty for *Paris* et al. (57) is low, with a NOS score of 7 and a GRADE rating of 3, whereas the other study (60) has a high risk of bias (NOS score of 3) and moderate certainty (GRADE: 6). In addition to fatty acids, alterations in phospholipids have also been observed, with elevated levels of PE, a major determinant of the membrane integrity and mitochondrial fitness (60). Changes in APO (61–64), or more specifically in APO 2 (61), were reported. Further studies found increases in APOA2 and APOA4 (62), as well as in APOD (63) and APOE (64).

Altogether, changes in fatty acid levels alter the TCA cycle, as fatty acids form acetyl-CoA through β-oxidation, thereby affecting energy metabolism.

#### 3.6.4 Inflammation pathways

**Figure 7:**
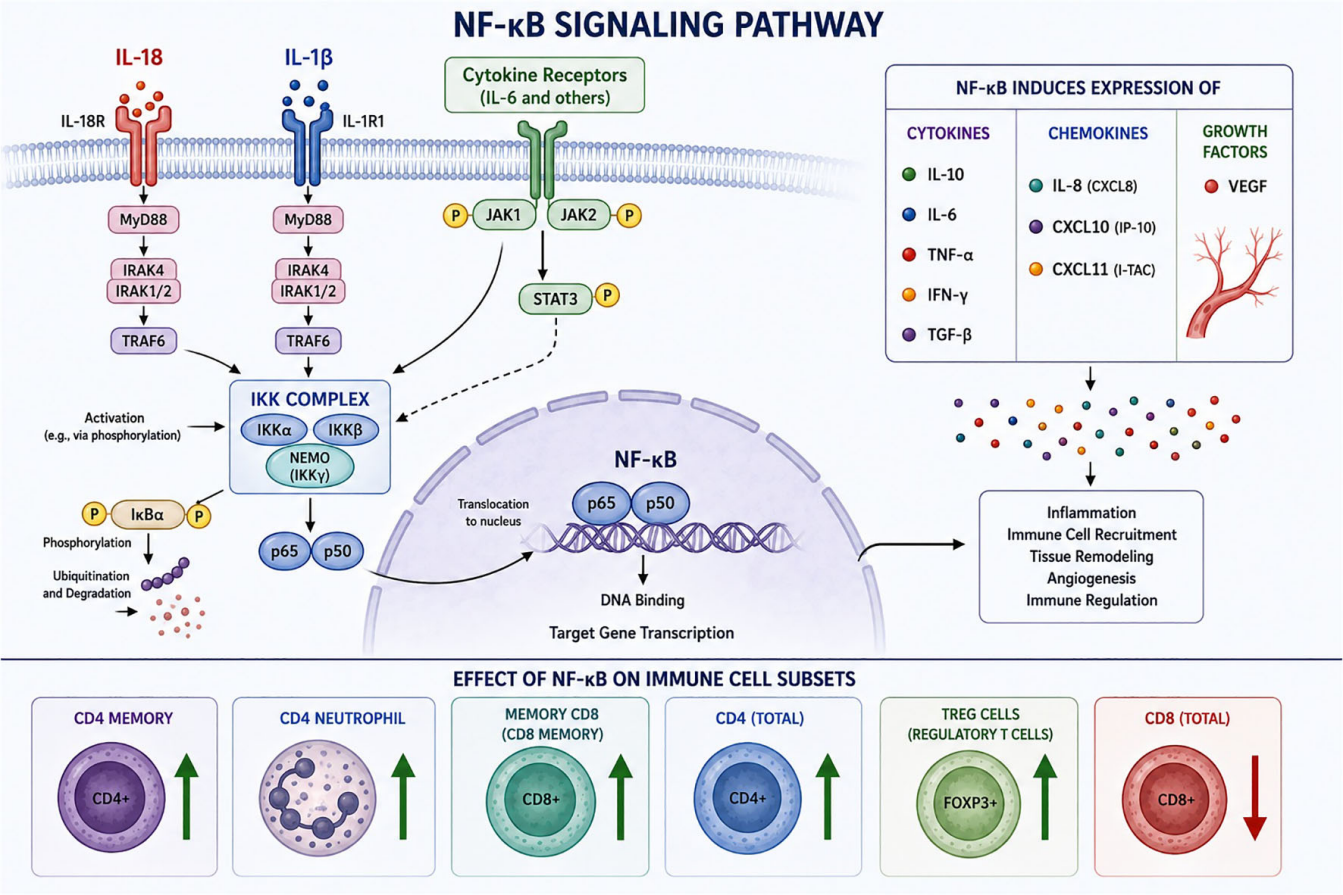
NF-κB signaling pathway and its potential immunological effects in post-acute inflammatory conditions. Schematic overview of NF-κB activation through IL-18-, IL-1β-, and cytokine receptor-mediated signaling pathways involving MyD88, IRAK1/4, TRAF6, IKK complex activation, and downstream NF-κB (p65/p50) nuclear translocation. The figure further illustrates the associated expression of inflammatory cytokines, chemokines, and growth factors, including IL-6, IL-8, IL-10, TNF-α, TGF-β, IFN-γ, CXCL10, CXCL11, and VEGF, as well as the potential influence of NF-κB signaling on immune cell subsets, including CD4 memory cells, neutrophils, memory CD8 cells, regulatory T cells (Treg), and total CD8 populations. Created with assistance from ChatGPT image generation (OpenAI, San Francisco, CA, USA).

In the context of NF-κB-related inflammatory signaling (**Figure 7**), findings suggest persistent immune activation, as reflected by elevated proinflammatory cytokines and complement components across several studies (49, 52, 53, 64–81). This pattern indicates that chronic immune engagement may, at least in part, be mediated through NF-κB-dependent pathways. Overall, the findings were consistent, showing widespread activation of chemokines and cytokines. Notably, increases in IL-18 (53, 65) and IL-6 (50, 54, 66–71) were observed, with IL-6 levels being reduced in response to probiotic treatment (72). Other studies have reported a decrease in IL-6 (73, 74), IFN-γ (74), and TNF-α (69, 74). Furthermore, elevated levels of TNF-α (65, 67, 75–77), IFN (67, 76, 78), and IL-8 (56, 71) have been reported, whereas IL-8 and TNF-α levels were reduced following beetroot supplementation (79). In contrast, *Chen* et al. reported a decrease in IL-8 (80). The contradictory findings cannot be explained solely by differences in risk of bias and certainty of evidence, since the majority of studies reporting opposing results showed a high risk of bias (NOS scores of 3-4) and moderate certainty (GRADE ratings of 5-6) (50, 54, 66, 71). The exception was one study (68), which achieved an NOS score of 6, indicating a moderate risk of bias and good certainty, with a rating of 4. Similarly, two studies with moderate certainty ratings of 5 and 7, differing in their methodological quality (74, 75), demonstrated inconsistent findings: one study showed a high risk of bias (74), and the other a moderate risk (75). Therefore, factors beyond methodological quality are likely contributing to the observed discrepancies. Additionally, CXCL10 levels were elevated (65, 81), whereas CXCL11 levels were decreased (81, 82).

**Figure 8:**
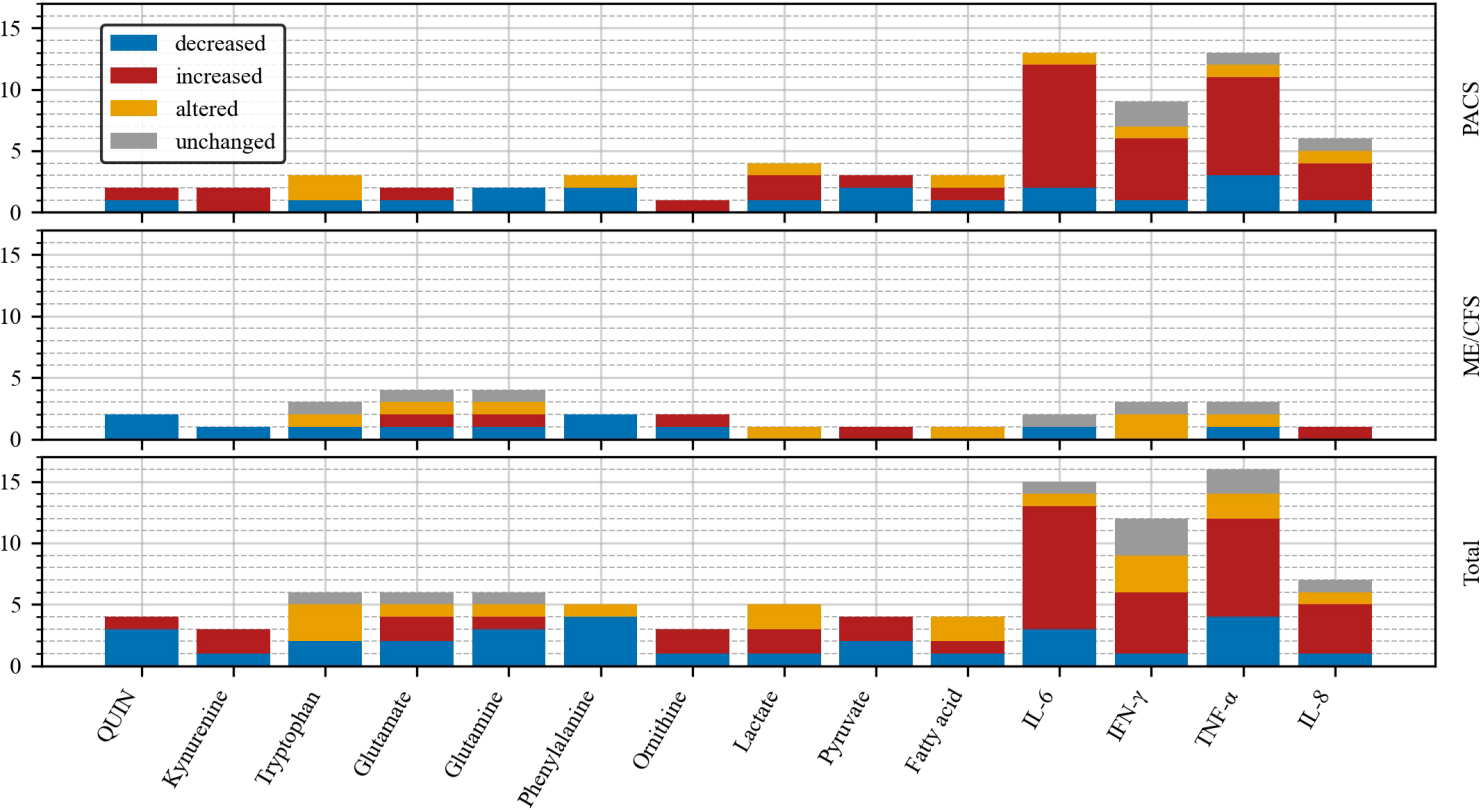
Comparative distribution of altered biomarkers across PACS and ME/CFS studies. Stacked bar charts illustrating the number of studies reporting decreased, increased, altered, or unchanged levels of selected metabolites, cytokines, and inflammatory markers in post-acute COVID syndrome (PACS), myalgic encephalomyelitis/chronic fatigue syndrome (ME/CFS), and the combined total dataset. Biomarkers include metabolites associated with the kynurenine pathway and energy metabolism (QUIN, kynurenine, tryptophan, glutamate, glutamine, phenylalanine, ornithine, lactate, pyruvate, and fatty acids), as well as inflammatory mediators (IL-6, IFN-γ, TNF-α, and IL-8). The figure highlights substantial heterogeneity among studies but shows a predominance of elevated pro-inflammatory cytokines, particularly IL-6 and TNF-α, in both PACS and ME/CFS cohorts.

In general, NF-κB is activated by factors such as infections and Toll-like receptor (TLR) signaling, leading to increased levels of cytokines (IL-6, IL-8, IL-18, TNF-α, IFN) and chemokines (CXCL10, CXCL11). The increase in IL-18 and TNF-α activates NF-κB (positive feedback loop), as well as IL-6 and IL-8. The activation of NF-κB thus leads to further activation of IFN-γ. IFN-γ activates CXCL10 and CXCL11 but simultaneously inhibits NF-κB, leading to decreased levels of NF-κB and of cytokines and chemokines. Furthermore, IFN-γ activates T cells, thereby linking the signal transduction level with the cellular level (**Figure 8**).

Further immune-related findings were observed at the cellular level, including increases in overall T cells, regulatory T cells (TREG) (84), and cytotoxic and activated memory CD4^+^ and CD8^+^ T cells (38, 78), suggesting ongoing immune activation. For naïve T cells, conflicting results have been observed, where decreases (76, 83), increases (84), and no differences between groups for T cells in general were reported (85, 86). In contrast, memory T cells (both CD4^+^ and CD8^+^) showed consistent findings, with increases reported across multiple studies (38, 76, 84) and potential sex-specific differences including elevations in females (76), although decreases in cytotoxic CD8^+^ T cells have also been reported in females (77). Additionally, neutrophil counts were elevated (38), further supporting sustained immune activation. Natural killer (NK) cells were generally decreased (38, 74, 87), but increases in males (77), and in comparison to convalescent individuals (89) were also reported. Significant differences were found between PACS and acute or healthy cohorts (85) but not recovered COVID-19 cohorts (85, 88). Only two studies (76, 86) have a moderate risk of bias (NOS score: 6 and 5) and good certainty of evidence (GRADE: both 4), while one study has high uncertainty with a GRADE rating of 8 (85).

Overall, the increase in memory T cells suggests prior antigen exposure and the formation of immunological memory, whereas the elevation of cytotoxic and activated T cell subsets indicates persistent immune activation. The observed reductions in total T cells and CD8^+^ T cells, together with alterations in NK cell levels, further point to immune dysregulation with potential sex-specific effects. The observed discrepancies cannot be attributed to differences in evidence level alone but are likely explained by the analysis of different T cell subpopulations, cohort selection, as well as by sex-specific differences (**Supplementary Table 4**).

#### 3.6.5 Mitochondrial dysfunction

Mitochondrial dysfunction emerged as another recurring feature of PACS. Multiple studies reported alterations in mitochondrial DNA (mtDNA) genes encoding components of the electron transport chain, including Complex I (MT-ND1, MT-ND2, MT-ND5, MT-ND6), Complex III (MTCYTB), Complex IV (MT-CO1), and Complex V (MTATP6) (90, 91). Collectively, these genes are central to oxidative phosphorylation and ATP production, suggesting that PACS is associated with disturbances in mitochondrial energy metabolism. The predominance of alterations affecting Complex I further points to dysregulation of NADH-dependent electron transport, potentially contributing to impaired cellular energy generation and altered redox homeostasis.

#### 3.6.6 Endothelial and vascular dysfunction

Endothelial and vascular dysfunction is also implicated, as evidenced by reduced levels of VEGF-A and ICAM-1 (92) and altered levels of VEGF-B (38), suggesting a shift from acute inflammation- and injury-related angiogenesis toward vascular protection and adaptive responses. In support of these findings, a change in VEGF-A levels was observed (75). In contrast, increased levels of ICAM-1 (76, 87), CRP (76, 87), SAA (76), VCAM-1 (87), and VWF (87), VEGF-A (89), and reduced levels of VEGF-C (76) were observed, indicative of impaired tissue repair.

Of the studies reporting contradictory results, *Shahbaz* et al. (76) achieved good evidence (GRADE: 4) and the highest NOS score (6), indicating a moderate risk of bias. This is similar to the classification of *Obe*r et al. (92) (NOS score: 4, GRADE: 4), which was also classified as having good evidence and a moderate risk of bias. In contrast, the studies by *Sykes* et al. (NOS score: 4, GRADE: 6) (87), *Farré* et al. (NOS score: 4, GRADE: 5) (89)*, Iosef* (NOS score: 4, GRADE: 7) (75) and *Shahbaz* et al. (38) (NOS score: 3, GRADE: 5) were classified as having a high risk of bias and moderate certainty of evidence. An increase in platelet activation (83, 93) was detected (94), pointing toward inflammation-driven vascular injury and dysregulated angiogenesis. A change in PF4 was observed in *Chakraborty* et al. (93).

In general, inflammatory cases are characterized by elevated levels of CRP, SAA, ICAM-1, VCAM-1, and VWF. This increase is a response to endothelial damage, which in turn releases VWF, ICAM-1, and VCAM-1. This results in reduced VEGF-A and VEGF-C levels, thereby stimulating angiogenesis and lymphangiogenesis, indicating impaired endothelial and vascular function.

Consistent with these findings, activation of the complement system was observed. Direct influence was reflected in increased CD55 levels, which protect healthy cells from the immune system (95), as well as alterations in the levels of the C1 inhibitors C3, which activates the adaptive immune system, and C5, which prevents autoimmune reactions (94). Indirect effects on the complement cascade are associated with changes in *COMP*, *COL1A1*, and *FLNA*, affecting structural integrity, cell shape and migration, and activation and migration of leukocytes*, and PLG, SERPINE1*, and *SRGN*, affecting tissue remodeling, blood clotting and cell migration and adhesion, and secretion of inflammatory mediators (64).

#### 3.6.7 Gene expression levels

Genomic, epigenetic, and transcriptomic alterations have also been reported. These include decreased expression of *LILRB1/2* (95) and differing levels of inflammation-associated microRNAs, including changes in miR-9-5p and miR-486-5p (96). Specifically, an increase in hsa-miR-146a-5p was detected, whereas the levels of hsa-miR-126-3p and hsa-miR-223-3p were reduced (57). An increase in hsa-miR-1307-3p, as well as changes in hsa-miR-26a-5p and miR-186-5p, were observed (97). These results suggest complex immune regulation. On the one hand, an increase in hsa-miR-146a-5p indicates a potential downregulation of immune responses, while, on the other hand, a decrease in LILRB1/2, hsa-miR-126-3p, and hsa-miR-223-3p points toward elevated inflammation or excessive immune activation.

Within the miRNA network, these miRNAs interact, among other things, with SAA via TLR2/4; and with the olfactory receptor genes (OR), Gal-9, RELN, ARTN, COL1A1, and SERPINE. The TLR2 level was elevated (75) and could be reduced by parabiotic treatment (98). This elevation, in turn, leads to an increase in SAA in general (63) and particularly in men and women (76). Changes in OR were also described by *Ray* et al. and *Shahbaz* et al. (74, 76). A decrease in OR1L8, OR52H1, OMP, and OR2L5 was observed (74), whereas an increase in ORAF17, OR2L2, OR10K1, OR1A1, OR2T12, OR14J1, and OR5A1 was reported (76). Furthermore, sex-dependent differences were identified (76). In connection with PACS, an increase in galectin-9 was observed (76, 99), which is generally inhibited by OR genes, as well as increases in reelin (38, 76, 83) and ARTN (76, 99). Consistent with this, an increase in COL1A1 (64) and SERPINE (64, 100) was observed.

In summary, the increases in SAA, RELN, ARTN, COL1A1, SERPINE, GAL-9, and OR indicate systemic inflammation.

#### 3.6.8 Microbiome

Analysis of the microbiome revealed notable differences between ME/CFS and PACS, indicating disease-specific alterations in the gut microbial composition. Bacterial diversity (especially the Firmicutes phylum) (43) and eukaryotic diversity (45), as well as β-diversity, were reduced in ME/CFS, whereas α-diversity showed no change in PACS cohorts; neither α nor β diversity differed significantly, suggesting that overall microbial richness and community composition remain unchanged (101–103). β -diversity showed differences with respect to sex, particularly among Bacillota and Bacteroidota species (101).

**Figure 9:**
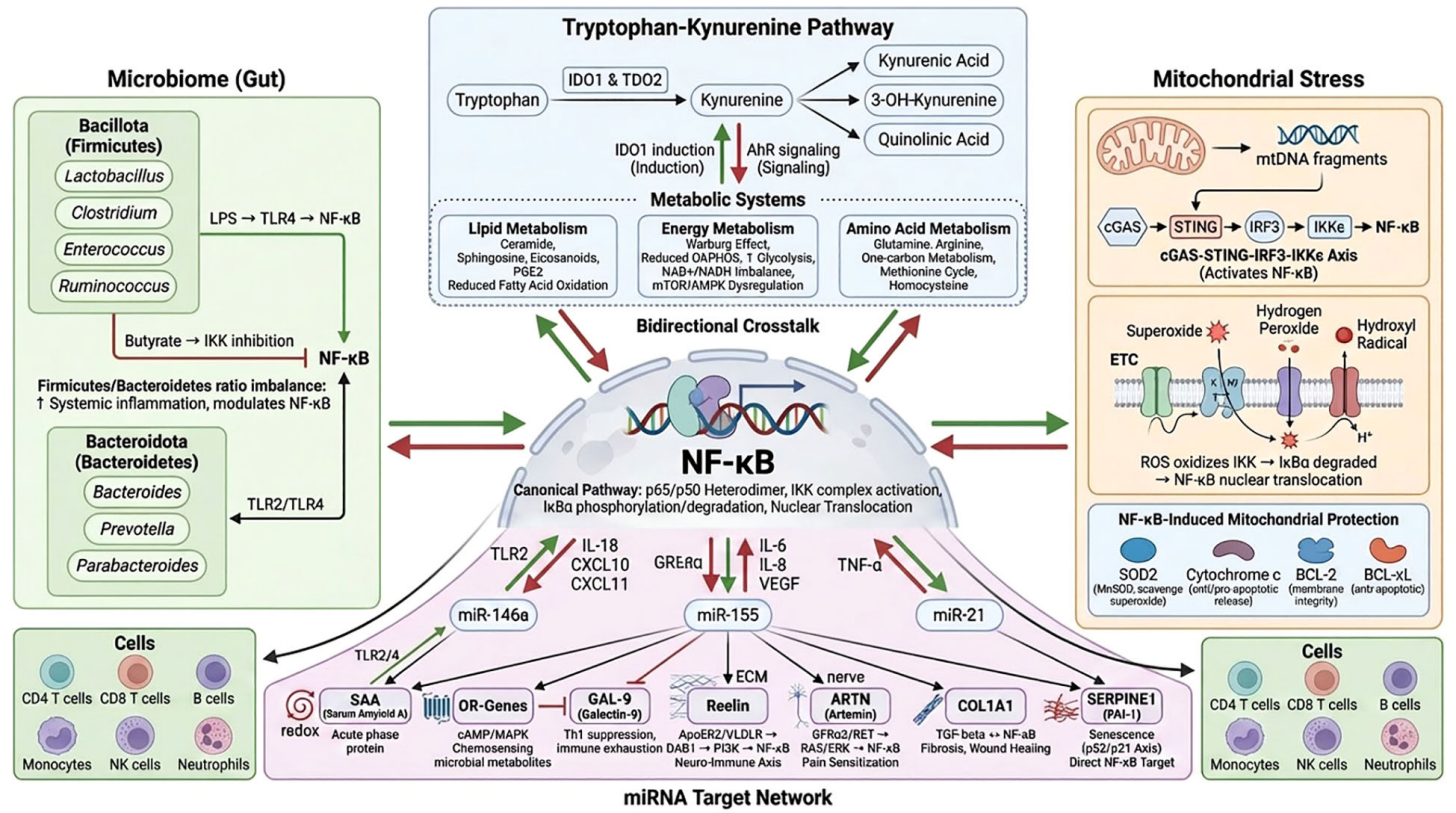
Integrated model of NF-κB-associated immunometabolic dysregulation involving the microbiome, kynurenine metabolism, mitochondrial stress, and miRNA signaling. Schematic overview illustrating the proposed central role of NF-κB in coordinating inflammatory, metabolic, and mitochondrial pathways potentially associated with post-acute infection syndromes. The figure shows the kynurenine pathway, mitochondrial stress responses mediated via the cGAS-STING-IRF3-IKKϵ axis, reactive oxygen species (ROS) production, and NF-κB-dependent cytokine expression. Furthermore, the diagram summarizes potential downstream interactions with metabolic systems, miRNA-associated regulatory networks (including miR-146a, miR-155, and miR-21), extracellular matrix remodeling, neuroimmune signaling, and immune cell alterations involving CD4 T cells, CD8 T cells, monocytes, NK cells, B cells, and neutrophils. Green and red arrows indicate activating and inhibitory interactions, respectively. Created using FigureLabsAI (FigureLabsAI platform, 2026). License ID: FL-PUB-20260506-S1M8A0.

In summary, a shift in the balance between the Bacillota and Bacteroidota can lead to an increase in systemic inflammation and modulate the NF-κB pathway (**Figure 9**).

#### 3.6.9 Sex-specific difference

Sex-based comparisons were conducted in seven studies. Significantly fewer differences were observed in the microbiome of men than in that of women. A change in the beneficial Bacteroidota and Eubacterium, as well as a decrease in Bacteroidota and an increase in Bacillota, was observed (101). By contrast, the microbial taxa observed in women showed both increases and decreases, including beneficial and opportunistic characteristics (101). Increased abundances were observed for the butyrate-producing genera Agathobacter and Fusicatenibacter (101), which are associated with immune system regulation and anti-inflammatory effects. Furthermore, the increased presence of Blautia, Ruminococcus, and Ruminococcaceae (103) indicates an enhanced ability to produce short-chain fatty acids and ferment fiber. Conversely, reductions were observed in beneficial taxa, including Lachnospiraceae and Bacteroides (101), which are involved in butyrate production, complex carbohydrate degradation, and generation of immunomodulatory metabolites. There were also reductions in the ambivalent taxon Lachnoclostridium (101). At the same time, several opportunistic or potentially pro-inflammatory taxa, including Enterobacteriaceae, Escherichia/Shigella, Streptococcus, Veillonella, and Peptostreptococcus, decreased in abundance (101).

In *Shahbaz* et al., sex-dependent differences were analyzed at the single-cell and bulk RNA levels. In women, this included increases in INF-γ, VCAM-1, CXCL-8, GZMA, and ORs, and decreases in IL-16, eotaxin-3, TGF-β1, and testosterone. In men, however, there was an increase in ARTN and a decrease in CXCR3 and estradiol (76). *Feliz* et al. observed a decrease in CD8^+^ cytotoxic T cells in women, whereas in men, increases in TNF-α, NK cell function, B cells, and humoral immunity were observed (77).

The imbalance in the microbiome also increases systemic inflammation in both sexes. In women, the inflammatory process is activated. The increase in IFN-γ leads to an inflammatory loop, increasing VCAM-1, CXCL8, and GZMA, and is a sign of acute inflammation. The reduction in TGF-β1 and testosterone reduces the inhibition of IFN-γ and CD8 cells, which is why there should be an increase in IFN-γ and CD8 T cells. NF-κB is activated in men. An increase in TNF-α leads to NF-κB activation, increased levels of the immunomodulatory ARTN, and enhanced NK cell and B cell function. It also inhibits estradiol, which in turn inhibits CXCR3, thereby suppressing the immune response.

A summary of the results at the molecular level, showing the predominant molecules, is presented in **Figure 8**.

### 3.7 GBS results

The findings from GBS were used as a mechanistic comparator to contextualize and compare the results observed in PACS and ME/CFS. GBS is an acute autoimmune disease characterized by rapid-onset peripheral neuropathy, immune-mediated nerve damage, and clinical features such as ascending paralysis, areflexia, and autonomic dysfunction. Due to the significant symptom overlap, particularly in fatigue, neurological deficits, and immune dysregulation, GBS serves as a relevant model for investigating shared pathophysiological mechanisms, including autoantibody-mediated damage, neuroinflammation, and post-infectious immune activation. This makes it a valuable mechanistic comparator for supporting biological interpretations.

A total of sixteen studies were identified in our search strategy (**Supplementary Table 5**), of which eleven were included in the analysis, and five studies were excluded (four reviews and one without a GBS focus). The average sample size across these studies was 38 ± 12 participants. Three studies compared GBS with multiple sclerosis (MS), three with chronic inflammatory demyelinating polyneuropathy (CIDP), one with non-neurodegenerative neurological diseases (NND), and one with spinal cord injuries (SCI).

#### 3.7.1 Study characteristics

In terms of the sample materials used, seven studies analyzed blood samples (163–171), including serum, plasma, or peripheral venous blood, three studies examined CSF samples (169–171), and two studies combined both sample types (163, 165). A total of eight proteomics, three metabolomics, and two transcriptomics analyses were performed in the included studies, with two of these metabolomics studies specifically including lipidomics components.

The analytical methods varied across the studies, reflecting methodological heterogeneity. In the field of proteomics, MALDI-TOF analyses and two-dimensional gel electrophoresis (2DE) have been used in two studies, and tandem mass spectrometry (MS/MS) in one. Highly sensitive analytical approaches such as shotgun mass spectrometry and stable isotope dilution multiple reaction monitoring (SID-MRM) had been applied in one study to quantify proteins. Proximity extension assays (PEA) using Olink^®^ multiplex panels had been employed in another study, whereas ELISA-based methods had been used in one study for targeted protein detection. Electrophoretic techniques applied in a single study had been used to separate proteins. In addition, computer-assisted approaches, including Bayesian networks and other machine learning methods, had been utilized in one study to identify and classify complex protein or metabolite signatures.

#### 3.7.2 Key molecule results

When GBS was compared with PAIS, alterations were observed across multiple functional systems, including amino acid, energy, and lipid metabolism, as well as immune-related pathways (**Table 3**). Changes in amino acid metabolism, particularly within the branched-chain amino acid (BCAA) pathway, were characterized by alterations in leucine and decreased methionine levels (171). In addition, reduced creatine levels may further reflect disturbances in the creatine-phosphate system and energy metabolism (171).

**Table 3:** Shared and distinct biomarkers in PACS, ME/CFS, and GBS-stratified by functional systems.

Functional system	Shared biomarkers (GBS & PACS/ME/CFS)	Biomarkers reported only in GBS
Immune system/Inflammation/Endothelium	Complement system, ITGA, NRP, Haptoglobin, Fibrinogen, ICAM, IL-17, IL-6	IL-2RA, SELE, Piccolo
Lipid metabolism/Membrane structure	Ceramide, Apolipoproteins, Cholesteryl esters, PC (phosphatidylcholine), Ethanolamine	LysoPC, Transthyretin, Albumin
Energy metabolism & mitochondrial reprogramming	Leucine, Methionine, Acetate, Methanol, Creatine, Glucose	Acetoacetate, 3-Hydroxybutyrate, Monoacetylglycerate, Glycolytic acetyl, Isobutyrate, Fructose, Threose
Neuro-immune signaling/Cell adhesion	ICAM, ITGA, NRP	NCAM1
Erythropoiesis/Oxygen transport	/	Albumin, Transthyretin
Carbohydrate & one-carbon metabolism	Choline	Fructose, Threose
Phospholipid and membrane metabolism	PC, CCM	LysoPC
Acute-phase and inflammatory proteins	Haptoglobin, Fibrinogen	Transthyretin, Albumin

Alterations in lipid-related metabolites were observed, including increased levels of cholesterol esters, phosphatidylcholine (PC), sphingomyelin (SM), lysophosphatidylethanolamine, and lysophosphatidylcholine (LPC). Increased levels of the sphingolipid ceramide (Cer) were also reported In one study, LPC and acylcarnitine showed potential discriminatory value for distinguishing GBS from comparison groups (166). At the protein level, decreased apolipoprotein E (163, 170) and increased apolipoproteins A-IV (170), H (163), and C3 (169) were observed.

Several studies reported differences in immune- and inflammation-related markers, including elevated levels of IL-8, IL-2RA, IL-6, SELE, ITGAM, and NRP1 (163), while ICAM-1 and IL-17 were additionally reported as altered (164). The complement component C3 isoform was increased (163), together with elevated haptoglobin levels (170). Furthermore, albumin (Pro2044) was increased, whereas fibrinogen was decreased (170). The adhesion molecule NCAM-1 was reduced (169), and the presynaptic protein piccolo showed altered expression (164). Transthyretin levels were decreased (163, 170), and reductions in albumin (163) and TGOLN2 (169) were also observed.

Additional metabolic alterations were reported, including increased acetoacetate, monoacylglycerols (1-monopalmitin and 1-monostearin), 3-hydroxyisobutyrate, and glycolic acid, while threose, methanol, and acetate were decreased. Additional alterations were reported for glucose, fructose, acetone, isobutyrate, and choline (171). In addition, *Zhang* et al. observed changes in CXCL1, NR3C1, and STAT3, among others (167), while *Lima* et al. demonstrated an increase in AIM2, NLR family genes, LRR protein genes, and IL-10 (168).

The adjusted GRADE analysis revealed that, of the 11 studies included, seven were rated with an initial score of 2, three with an initial score of 3, and one was a pilot study (score 5) (**Supplementary Table 5**). Due to the inclusion of penalties, all studies are subject to a moderate degree of uncertainty. Using the Newcastle-Ottawa Scale (NOS) (**Supplementary Table 5**), nine studies were classified as having a high risk of bias (163–172), one study (173) was rated as having a moderate risk of bias, and one (165) as having a low risk of bias. After accounting for penalties, all 11 studies ultimately showed moderate certainty. In general, the studies had a small sample size but good directness of evidence.

Comparative analysis of GBS and PAIS, including PACS and ME/CFS, revealed both shared and distinct biomarker profiles across these functional systems (**Table 3**). Shared alterations were observed in amino acid metabolism, lipid metabolism, and immune-related markers, whereas condition-specific differences were mainly evident in phospholipid species and cytokine patterns.

## 4 Discussion

This discussion is structured in several sections to contextualize the findings of this systematic review. First, we address the major limitations of the included studies and the resulting constraints on the interpretation of the evidence, particularly regarding sample size, methodological heterogeneity, and variability in analytical approaches. Second, we present a proposed pathophysiological framework that integrates the identified biomarkers and biological pathways from the current literature. This framework aims to connect the observed multi-omics alterations and inflammatory processes into a broader mechanistic model of post-acute infection syndromes (PAIS). We further discuss potential feedback loops and interacting processes that may contribute to persistent disease states. In addition, we briefly compare PAIS with Guillain-Barré syndrome (GBS), particularly regarding potentially shared post-infectious mechanisms and the ongoing question of whether GBS may represent a specific manifestation within the broader PAIS spectrum. Finally, we position our review within previous reviews in the field and highlight the distinguishing aspects of our approach, including the integration of multi-omics findings, biomarker heterogeneity, and mechanistic pathway interpretation across diverse PAIS-related conditions.

### 4.1 Review limitations

This systematic review synthesized biomarker-related findings across PAIS. The majority of papers analyzed PACS and ME/CFS, whereas biomarker research in other PAIS is still emerging. Other PAIS entities remain underrepresented or unstudied at the molecular level, underscoring a substantial research gap. This imbalance may partly reflect the search strategy, which explicitly targeted PACS, ME/CFS, and post-Lyme disease through specific MeSH terms. In contrast, other PAIS were captured only by broader, nonspecific terms, potentially leading to their underrepresentation. The varying definitions of PACS (Post-COVID, long COVID, and related terms), along with differences in terminology and diagnostic criteria, may also significantly influence study selection and search results. The absence of protocol registration constitutes a methodological limitation, as it reduces transparency and hampers the assessment of potential reporting bias.

Studies explicitly focusing on EBV and CMV were not found in our search. In the retrieved ME/CFS studies, multiple samples tested positive for prior infection, suggesting that EBV and CMV may be implicitly represented within ME/CFS cohorts.

The inclusion of GBS may be subject to debate, as its classification within a PAIS framework remains unresolved. However, it was incorporated to facilitate mechanistic comparison across post-infectious conditions.

### 4.2 Data/studies’ limitations

A key limitation identified in this review is the pronounced geographic imbalance in the study populations. While cohorts from North America and Europe were frequently analyzed, regions such as Australia and Africa were markedly underrepresented. This skewed regional representation reduces the generalizability of findings and introduces potential for systematic bias. Given the influence of environmental, genetic, and socioeconomic factors on disease development and progression, the lack of global data coverage might limit the external validity of biomarker findings in PAIS. Proactive consideration of demographic diversity, including balanced representation of sexes, age ranges, and geographic regions, is essential for establishing robust, broadly applicable biomarker panels for post-acute infection syndromes.

The methodological landscape of the included studies was considerably heterogeneous. Proteomic analyses were the most prominent omics approach (45.1%), but techniques varied widely across studies. While cross-study comparisons are theoretically possible, the selective focus on predefined molecular targets in many investigations hinders comparability. Because different sets of molecules were analyzed in each study, overlapping findings may be coincidental rather than indicative of consistent biological signatures. On the other hand, the results are more likely to be reliable when they are confirmed via different methods. The observed variability in reported biomarker levels across studies may be attributed to several interrelated methodological and clinical factors. First, the analytical platforms used differ in sensitivity, specificity, and dynamic range (**Table 4**). As a result, some assays may fail to detect subtle alterations in biomarker concentrations, leading to apparent inconsistencies between studies. For instance, low-abundance proteins may remain undetected in ELISA- or NMR-based metabolomics assays. In contrast, mass spectrometry or multiplexed proteomic panels can capture a broader spectrum of molecules, thereby directly affecting the reported biomarker profiles. Second, tissue specificity plays an important role. While the majority of studies analyzed blood samples, other matrices were used in a subset of studies, including fecal samples, urine, cerebrospinal fluid, and single studies analyzing lung tissue, bronchoalveolar lavage fluid (BALF), EBC, or ccf-mtDNA. Different tissues may reflect distinct pathophysiological aspects, limiting the comparability of biomarker levels across studies. Finally, variability in patient selection further contributes to heterogeneity. As shown in **Supplementary Table 3**, studies differed in how the control and test groups were defined, resulting in a wide range of inclusion criteria and comparator populations. Combined, these factors, mainly assay characteristics, symptom severity, tissue source, and patient selection, may explain why biomarker results often differ between studies. They also highlight the urgent need for standardized definitions and protocols for sample collection, patient selection, and biomarker measurement, to enable meaningful cross-study comparisons and analyses.

**Table 4:** Comparative summary of methods included a comparison of sensitivity, specificity, range, and impact on biomarkers.

Platform	Sensitivity	Specificity	Dynamic range	Impact on reported biomarkers
Mass spectrometry	High (fM-nM)	High	Very wide (~10^6^-10^8^-fold)	Enables quantification of many metabolites/proteins simultaneously; analytical variability possible
ELISA	Medium-high (pg/mL-ng/mL)	High	Narrow (~10²-10³-fold)	Targets individual proteins: results can vary by kit; potential cross-reactivity
Olink Proteomics Panel	High (pg/mL)	High	Moderate (~10²-10³-fold)	Multiplexed protein detection; relative quantification; limited absolute concentration values
Flow cytometry	Medium-high (marker-dependent)	High	Moderate (~10²-10³-fold)	Cell-specific expression; depends on antibody quality; rarely provides absolute concentrations
NMR-based metabolomics	Medium (µM-mM)	High	Narrow (~10¹-10²-fold)	Detects mainly high-concentration metabolites; low sensitivity for trace metabolites
Machine learning in LMM	Variable (model-dependent)	Variable	Model-dependent	Detect patterns or combinations; sensitivity and significance depend on the dataset and preprocessing
Western blot	Medium (ng)	High	Narrow (~10²-fold)	Semiquantitative; depends on antibody and sample quality; single protein measurement

The evidence table (**Supplementary Table 1**) demonstrates that the majority of included studies were prospective cohort studies. This distribution is consistent with the overall study aim, as the included literature predominantly compares patient groups (e.g., PACS, ME/CFS, or related post-infectious syndromes) with control groups (e.g., healthy or recovered individuals), rather than evaluating interventional effects. Consequently, the highest level of evidence in the traditional hierarchy (i.e., intervention-based randomized controlled trials) is not the primary study design in this field. The predominance of cohort-based designs is therefore expected and reflects the observational nature of post-acute infection research. In particular, retrospective cohort studies may contribute valuable insights due to their often-larger sample sizes, which increase statistical power and enable the detection of subtle biological effects. This is especially relevant in post-acute infection syndromes, where biomarker profiles remain largely exploratory and not yet fully validated. Accordingly, retrospective analyses with broad patient cohorts provide an important exploratory foundation, especially for machine learning approaches. A key advantage of machine learning approaches is that they can operate without a predefined selection of molecules, in contrast to mass spectrometry, ELISA panels, flow cytometry, or Western blotting, which rely on targeted molecule selection.

A modified GRADE assessment revealed that the majority of the included studies (65.5%) were categorized as having moderate certainty of evidence. The most frequent factor contributing to downgrading was imprecision, primarily due to limited sample sizes. Therefore, larger, adequately powered studies are needed to substantially reduce uncertainty and strengthen the reliability of the reported findings. The Newcastle-Ottawa Scale (NOS) assessment revealed that most of the included studies were categorized as having moderate to poor methodological quality, indicating a moderate to high risk of bias. Only a small number of studies exhibited a low overall risk of bias. These findings have important implications for interpreting the reported results, as an elevated risk of bias may reduce the reliability and validity of observed biomarker associations. Furthermore, inconsistent study quality and methodological weaknesses limit comparability across studies, complicating the interpretation of conflicting findings and reducing confidence in the overall evidence base. Of particular concern was the poor performance in the comparability domain, given that adequate matching or statistical adjustment for potential confounders is essential in biomarker-based studies, making it difficult to compare different studies and ensure reproducibility. This issue is especially relevant given that several of the included studies indicated sex-dependent differences that could affect biomarker profiles and study outcomes. Consequently, inadequate matching of key variables such as sex, age, comorbidities, or disease severity may have led to inconsistent findings across studies.

Another frequently observed issue was limited methodological transparency, with many studies providing only brief descriptions of recruitment strategies, matching procedures, or exposure/outcome ascertainment. Consequently, some quality criteria may have been met but not explicitly reported, resulting in conservative NOS ratings and introducing an unavoidable degree of subjective interpretation. To reduce the risk of bias, it is advisable to follow guidelines designed to mitigate this risk and to register studies in advance, because studies can only be validated, compared, and reproduced if they are based on a transparent and easy-to-follow methodological framework. While most of the included prospective studies were registered, the issue remains, highlighting the necessity of risk-of-bias analysis by the study authors themselves when planning the study.

In addition to sex distribution and the magnitude of immune and metabolic alterations, sample size represents a critical factor influencing study outcomes. The statistical power of a study is largely determined by the number of participants, and smaller cohorts are less likely to detect subtle, but potentially meaningful, effects as significant. Many of the studies included in our analysis relied on relatively small sample sizes (**Figure 2C**), which likely contributes to the variability and occasional contradictions observed in biomarker levels and immune signatures across cohorts. This emphasizes the need for adequately powered studies to characterize sex-specific differences in post-acute infection syndromes reliably. The comparability of biomarker data across studies must be considered when interpreting the results, including small cohort sizes, the absence of age- and sex-matched controls, and the high diversity of assay platforms used. Therefore, findings remain preliminary and require validation in larger, longitudinal studies employing standardized methodologies and well-matched control groups.

### 4.3 Hypotheses

Despite this variability, consistent evidence points toward fundamental dysregulation of metabolic and inflammatory pathways in both PACS patients and ME/CFS patients. Shared observations include altered energy metabolism, amino acid imbalances (e.g., glutamine, kynurenine, glutamate), and immune system dysfunction. In accordance with our findings and their involvement in potential neurotoxicity, we propose central roles of QUIN, TKP, and KYNA in impairing energy metabolism. The observed reduction of QUIN in ME/CFS, a precursor to NAD+, could lead to NAD+ deficiency and, consequently, to cytoskeletal dysfunction (178). An increase in QUIN would promote the development of neurodegenerative diseases (174), but a reduction would also lead to an imbalance in the TKP and thus not result in reduced neurotoxicity. Furthermore, reduced TKR may not only lead to lower levels of QUIN but also to lower levels of serotonin, which in turn could lead to lower levels of melatonin, thereby impairing sleep regulation (174). TRP thus could have a direct influence on recovery, in addition to its effect on the balance between neuroprotection and toxicity. The results show a shift in TKP towards KYNA, whilst the neurotoxic pathway via QUIN is reduced. This shift is likely catalyzed by indoleamine-2,3-dioxygenase (IDO), which can be activated by IFN-γ (174) or LPS and potentially lead to a reduction in TKR, which is considered a hallmark of inflammation. QUIN, as a precursor of NAD^+^, has been shown to possess potent neurotoxic properties. It can act as a selective agonist of the N-methyl-D-aspartate (NMDA) receptor, and this binding could induce neuronal damage through a massive influx of calcium ions (175). Although KYNA is neuroprotective, elevated levels could also lead to pathological conditions (175). This could be explained by the dual function of TKP molecules: Under physiological conditions, KYNA is neuroprotective, but when its levels rise, it can act as a pro-oxidant and potentially lead to oxidative damage (174). Since NAD metabolism and TKP are closely linked and NAD metabolism and mitochondrial dysfunction are considered to go hand in hand with telomere shortening and cellular senescence as a prototypical mechanism of chronic diseases (174), TKP can also be directly linked to chronic disease. Furthermore, immunometabolic reprogramming, characterized by a metabolic shift toward glycolysis (176), is also considered a hallmark of activated immune cells and is frequently observed in infections (176). This is reflected in our findings in the elevated lactate (52, 57) and pyruvate levels (28, 54, 56), alongside reduced glutamine concentrations (30, 50, 54). Notably, these metabolic signatures appear to contrast sharply with those observed during acute SARS-CoV-2 infection, in which decreases in lactate and pyruvate, and elevations in glutamine are frequently reported (53). This divergence may reflect a fundamental difference between acute inflammatory responses and the chronic metabolic adaptation observed in post-acute sequelae. The persistent glycolytic phenotype could indicate sustained immune activation rather than a transient inflammatory burst and may contribute to cellular exhaustion and tissue dysfunction.

Furthermore, the potential role of a triggered innate immune activation and dysregulation is already well established in PAIS (1). Based on our findings, we propose several key mediators of the NF-κB pathway as central to this process: IL-6, TNF-α, CXCL10, and IFN-γ, which drive prolonged activation of neutrophils, macrophages, and natural killer (NK) cells (50). In this context, galectin-9 could emerge as a pivotal molecule, secreted predominantly by activated neutrophils. Its serum levels are positively associated with established inflammatory markers, such as CRP and IL-6 (179) and could potentially correlate with disease severity (180). Thus, we propose that galectin-9 functions could be a critical nexus, bridging classical inflammatory pathways with broader immunoregulatory mechanisms, including T-cell exhaustion via Tim-3 engagement (86), and amplifying systemic immune activation. This process could further corroborate the systemic impact of sustained inflammation on hematopoiesis. Evidence of erythropoietic dysfunction in our findings includes altered transferrin receptor expression (21, 38), altered hemoglobin levels (38, 42), and paradoxical increases in ferritin and lactoferrin, despite decreased serum transferrin. Erythropoiesis dysregulation is a well-established concept during acute SARS-CoV-2 infection (180) and PACS patients (50, 181), especially in females (76). These findings are consistent with anemia of chronic disease (ACD), where inflammation-driven iron sequestration impairs erythropoiesis. This iron redistribution could also be mediated via NF-κB-dependent pathways, linking hematological disturbances directly to the proposed overarching inflammatory network.

Notably, sex-dependent differences could be observed in post-acute infection syndromes, particularly in metabolomics studies. Mostly women, but not men, show alterations, for example, in IL-6, IL-12, IL-23, TNF-α, IFN-I, and chemokines, coupled with dysregulation of estrogen and testosterone, which influence inflammatory signaling, metabolism, and neuronal and cardiovascular function (76). We hypothesize that these findings may be clinically linked to greater neuroinflammation and cognitive dysfunction in women, whereas men might exhibit greater innate immune activation and mitochondrial stress (76). Importantly, variable female representation across studies likely contributes to conflicting results. For example, differences in lactate, fatty acid, and TNF-α levels correspond to cohorts with different proportions of female participants.

In addition to immune dysfunction, evidence of viral persistence has been found in COVID-19 (1). This process may involve the persistence of antigenic triggers, including viral reservoirs and dysregulated immune memory responses. Contrary to the initial hypothesis posited in the introduction, active viral replication appears to play a subordinate role in PACS pathogenesis. To date, no reproducible evidence of replicating SARS-CoV-2 has been reported in PACS cohorts (182), and the majority of studies included in this systematic review did not report any indication of active viral replication. Instead, the data suggests that chronic immune activation may not be driven by ongoing viral replication, but rather by persistent antigenic stimulation, potentially from viral protein fragments, immune complexes, or aberrant immune memory, supporting a shift from infectious persistence to immunological dysregulation.

Besides immune activation and viral persistence, indications of autoimmune activation and dysregulation of microbiome were observed in connection with an inability to repair damage (1). We propose that this condition may be linked to clinical symptom clusters via galactin-9, artemin (ARTN), and neutrophil extracellular traps (NETs). Elevated galectin-9 levels may be associated with fatigue, cognitive impairment, and neuroinflammatory processes (76, 99, 178). In contrast, ARTN, may indicate a possible association with pain syndromes, autonomic dysfunction (e.g., POTS-like symptoms), and sleep disturbances. Furthermore, NETs can be mechanistically linked to microthrombosis, endothelial damage, and organ-level complications. These correlations further support that the identified signaling pathways are not merely biochemical footprints but likely contribute directly to the pathogenesis of distinct symptom clusters, reinforcing the biological plausibility of a multisystem disease.

In line with these processes, *Trautmann* proposes four self-sustaining inflammatory loops, which, like our hypotheses, also center on IL-6 and TNF-α (183). The first loop involves T cells, B cells, and monocytes, i.e., the immune system. The second loop involves the production of inflammatory cytokines such as IL-6, TNF-α, and IL-1β by microglial cells. The third loop is based on unbuffered reactive oxygen species (ROS) damaging the mitochondria and leading to reduced ATP production (corresponding to mitochondrial reprogramming). The fourth loop involves bacterial translocation, leading to damage to the gut mucosa, which in turn can influence the microglial-gut-brain axis, thus affecting the neuroendocrine system (183). Thus, with regard to PAIS, there are various approaches to explain the disease that repeatedly return to the same core statements and key markers yet are based on complex and wide-ranging changes. Consequently, even reactions that appear contradictory when considered individually can, taken as a whole, lead to the same outcome: a dysregulated immune system with persistent symptoms.

Across all investigated domains, including metabolomic pathways (tryptophan-kynurenine metabolism, lipid metabolism, energy metabolism, and amino acid metabolism), the microbiome, mitochondrial stress (mtDNA fragments), and miRNA-target networks (including miRNAs, OR-Genes, GAL-9, ARTN, etc.), as well as cellular alterations in T and B cell compartments, a consistent link to the NF-κB signaling pathway seems to emerge. Therefore, we suggest that NF-κB may represent a central regulatory hub integrating multiple biological layers in post-acute infection syndromes. Within our proposed framework, diverse molecular mediators, including cytokines such as IL-6, IL-8, and TNF-α, as well as chemokines such as CXCL11 and CXCL11, appear to function as connecting nodes between otherwise distinct pathological domains. This interconnected signaling network could point toward the concept of a coherent, multi-system disease model (**Figure 9**), rather than isolated pathway disturbances. Overall, these findings appear to converge towards a unified pathophysiological framework, in which we propose that NF-κB-driven immune activation links metabolic dysregulation, immune cell alterations, and molecular stress responses across different biological systems. Taken together, the available evidence is consistent with a multi-system model involving interconnected biological pathways. However, the predominantly observational nature of the included studies and the moderate certainty of evidence preclude definitive conclusions regarding causality or the precise mechanistic role of NF-κB. Therefore, the proposed framework should be considered hypothesis-generating and requires further validation in mechanistic and longitudinal studies.

### 4.4 Comparison to GBS

In this review, GBS should primarily be interpreted as a mechanistic comparator rather than a core component of the PAIS spectrum. Its inclusion was intended to contextualize immune, inflammatory, and neurological alterations within a well-characterized post-infectious disorder, to identify potential converging mechanisms across post-infectious conditions, and to support biological interpretations of post-infectious diseases as well as PAIS-specific findings. The presented comparison of Guillain-Barré syndrome (GBS) and post-viral syndromes (ME-CFS and PACS) reveals both shared and disease-specific biomarker patterns, indicating overlapping yet distinct pathophysiological mechanisms. Across conditions, alterations were observed in amino acid metabolism, energy metabolism, lipid metabolism, and immune-related pathways, with both shared and distinct biomarkers identified within these functional systems. Taken together, these findings may support the concept of a shared immune-metabolic axis across post-infectious syndromes, characterized by ceramide accumulation, endothelial alterations, and cytokine imbalance. However, the opposing regulation of phospholipids (SM, LPC, PC) and divergent cytokine patterns (IL-6, IL-8) could underscore fundamental differences between acute immune-mediated neuropathy (GBS) and chronic post-acute infection syndromes (ME/CFS and PACS). Importantly, some of the observed differences may also be attributable to methodological heterogeneity between studies, as distinct analytical platforms and targeted biomarker panels were applied, potentially resulting in incomplete overlap of the measured biomarkers (**Table 3**). Given the similarities between GBS and PAIS, one might reconsider whether to classify GBS within the spectrum of PAIS or as a distinct clinical entity.

### 4.5 Comparison to other PAIS reviews

Among the specified search terms, 100 reviews were identified. However, none of these reviews addressed the full spectrum of PAIS in relation to omics. Instead, most studies have focused on a single PAIS and have typically analyzed only one type of omics data. This highlights the novelty and value of the present review, which integrates multiple PAIS conditions and combines multi-omics data, allowing for a more comprehensive understanding of shared and distinct mechanisms across syndromes. While some biomarkers have been reported in previous reviews, this systematic review integrates multiple omics and several PAIS, allowing the identification of similarities and differences across conditions and facilitating the detection of bias. Most existing publications focus on a single PAIS and a single omics type, whereas only five reviews address post-acute infection syndromes in general (1, 183–186), none of which include multi-omics analyses. This review, therefore, provides a comprehensive overview of PAIS representatives and integrates findings at the multi-omics level.

## 5 Conclusion

In this integrative review of state-of-the-art biomarkers in PAIS, we identified alterations in amino acid, energy, and lipid metabolism as well as changes in the microbiome, mitochondrial stress, and the miRNA target network. All of these changes are directly and indirectly linked to the NF-κB pathway. The main altered molecules included IL-6, TNF, and IFN.

Our analysis revealed widespread disruptions across amino acid-related metabolic pathways, energy metabolism, lipid metabolism, and immune signaling in post-acute infection syndromes, including ME/CFS and PACS. Alterations in the kynurenine pathway (kynurenine, tryptophan, kynurenic acid) and glutamate/glutamine metabolism suggest contributions to neurotoxicity, immune dysregulation, and inflammation, while perturbations in the phenylalanine-tyrosine and urea cycle pathways (ornithine, arginine, urea) may affect neurotransmitter balance and cognitive function. Changes in branched-chain amino acids (leucine, valine, isoleucine) and histidine likely impact muscle metabolism and further exacerbate immune dysregulation, whereas modifications in cysteine and methionine reflect increased oxidative stress.

Disruptions in energy metabolism (glucose, pyruvate, lactate, ATP) and lipid metabolism, particularly phospholipids and phosphatidylethanolamine, indicate impaired mitochondrial function, membrane instability, and altered signaling, which collectively may drive fatigue, reduced exercise tolerance, and systemic inflammation. Central mediators, including IL-6, TNF-α, NF-κB, and galectin-9, integrate these alterations across erythropoietic, mitochondrial, immune, and neuroendocrine pathways, forming a coherent network that underlies the multisystemic phenotype of PAIS.

Together, these findings support a conceptual disease model in which metabolic, immune, and neuroendocrine dysregulation converge, providing a mechanistic framework that links molecular alterations to clinical symptoms. This integrative perspective may guide future biomarker validation, targeted diagnostics, and therapeutic strategies for patients with ME/CFS and PACS. The mechanistic comparison with GBS also revealed changes across multiple functional systems, including amino acid metabolism, energy metabolism, and immune-related pathways.

PACS and ME/CFS appear to share similar pathomechanisms, as evidenced by comparable symptoms and biological aberrations. To elucidate the precise mechanisms underlying these syndromes, targeted analyses of multi-omics datasets are essential. While most existing studies have focused on limited panels of selected molecules, some have applied omics approaches, which offer a more comprehensive view of molecular alterations across layers (e.g., genomics, proteomics, and metabolomics) and enable broader comparability and validation. Therefore, leveraging comprehensive multi-omics datasets is crucial for advancing biomarker discovery and understanding the pathomechanisms involved in these conditions.

## Data Availability

The original contributions presented in the study are included in the article/**Supplementary Material**. Further inquiries can be directed to the corresponding author.

## Glossary

2DE: Two-dimensional gel electrophoresis
3-HK: 3-hydroxykynurenine
ACD: Anemia of chronic disease
APO: Apolipoprotein
ARTN: Artemin
ATP: Adenosine triphosphate
BCAAs: Branched-chain amino acids
CCC: Canadian Consensus Criteria
ccf-mtDNA: Circulating cell-free mitochondrial DNA
CD: Complement Decay-Accelerating Factor
CD4+ TEM: CD4+ Effector
CIDP: Chronic inflammatory demyelinating polyneuropathy
CRP: C-reactive protein
CSF: Cerebrospinal fluid
CXCL: C-X-C motif chemokine ligand
DNA: Deoxyribonucleic acid
EBC: Exhaled breath condensate
EBV: Epstein-Barr virus
ELISA: enzyme-linked immunosorbent assay
GAL-9: Galectin-9
GBS: Guillain-Barré syndrome
GRADE: Grading of Recommendations Assessment, Development and Evaluation
IDO: Indoleamine-2,3-dioxygenase
IFN: Interferon
Ig: Immunoglobulin
IL: Interleukin
IOM: Institute of Medicine
KMO: Kynurenine 3-monooxygenase
KYNA: Kynurenic acid
LPC: Lysophosphatidylcholine
MALDI-TOF: Matrix-Assisted Laser Desorption/Ionization - Time of Flight
ME/CFS: Myalgic encephalomyelitis/chronic fatigue syndrome
miR; miRNA: MicroRNA
MS: Multiple Sclerosis
MS/MS: Tandem Mass Spectrometry
mtDNA/MT: mitochondrial DNA
NAD+: Nicotinamide adenine dinucleotide
NADH: Nicotinamide Adenine Dinucleotide Hydrogen
NETs: Neutrophil extracellular traps
NF-κB: Nuclear Factor kappa-light-chain-enhancer of activated B cells
NHS: National Health Service
NICE: National Institute for Health and Care Excellence
NK: Natural killer cells
NMDA: N-methyl-D-aspartate receptor
NMR: Nuclear magnetic resonance
NND: Non-neurodegenerative neurological disease
OR: Olfactory receptor
PACS: Post-Acute COVID Syndrome
PAIS: Post-acute infection syndromes
PASC: Post-acute sequelae of COVID-19
PARK7: Parkinson disease protein 7
PC: Phosphatidylcholine
PE: Phosphatidylethanolamine
PEA: Proximity extension assay
PEM: Post-exertional malaise
PICOS: Patient, Intervention, Comparison, Outcome, Study type
POTS: Postural Orthostatic Tachycardia Syndrome
PRISMA: Preferred Reporting Items for Systematic reviews and Meta-Analyses
PTLDS: Post-Treatment Lyme Disease Syndrome
QUIN: Quinolinic acid
RELN: Reelin
RNA: Ribonucleic acid
ROS: Reactive oxygen species
SAA: Serum Amyloid A
SCI: Spinal cord injury
SID-MRM: Stable Isotope Dilution-Multiple Reaction Monitoring
SM: Sphingomyelin
TCR: T-cell receptor
TFN: Tumor necrosis factor
TKP: Tryptophan-kynurenine pathway
TKR: Tryptophan-kynurenine ratio
TREG: Regulatory T cells
VCAM: Vascular cell adhesion protein
VEGF: Vascular Endothelial Growth Factor
VWF: Von-Willebrand-Faktor-Receptor
